# Genetic characterization and pathogenic potential of H10 avian influenza viruses isolated from live poultry markets in Bangladesh

**DOI:** 10.1038/s41598-018-29079-1

**Published:** 2018-07-16

**Authors:** Rabeh El-Shesheny, John Franks, Bindumadhav M. Marathe, M. Kamrul Hasan, Mohammed M. Feeroz, Scott Krauss, Peter Vogel, Pamela McKenzie, Richard J. Webby, Robert G. Webster

**Affiliations:** 1 Department of Infectious Diseases, St. Jude Children’s Research Hospital, Memphis, TN 38105 USA; 2 Center of Scientific Excellence for Influenza Viruses, National Research Centre, Giza, Egypt; 3 Department of Zoology, Jahangirnagar University, Dhaka, 1342 Bangladesh; 4 Department of Pathology, St Jude Children’s Research Hospital, Memphis, TN 38105 USA

## Abstract

Fatal human cases of avian-origin H10N8 influenza virus infections have raised concern about their potential for human-to-human transmission. H10 subtype avian influenza viruses (AIVs) have been isolated from wild and domestic aquatic birds across Eurasia and North America. We isolated eight H10 AIVs (four H10N7, two H10N9, one H10N1, and one H10N6) from live poultry markets in Bangladesh. Genetic analyses demonstrated that all eight isolates belong to the Eurasian lineage. HA phylogenetic and antigenic analyses indicated that two antigenically distinct groups of H10 AIVs are circulating in Bangladeshi live poultry markets. We evaluated the virulence of four representative H10 AIV strains in DBA/2J mice and found that they replicated efficiently in mice without prior adaptation. Moreover, H10N6 and H10N1 AIVs caused high mortality with systemic dissemination. These results indicate that H10 AIVs pose a potential threat to human health and the mechanisms of their transmissibility should be elucidated.

## Introduction

Avian influenza viruses (AIVs) pose continual challenges to human and animal health worldwide. AIVs belong to the family *Orthomyxoviridae* and are classified on the basis of their surface glycoproteins hemagglutinin (HA) and neuraminidase (NA). Currently, 18 HA subtypes (H1–H18) and 11 NA subtypes (N1–N11) have been identified and, with exception of the H17N10 and H18N11 subtypes found in bats, all AIV subtypes are found in aquatic birds^1–4^. Only subtypes H1, H2, and H3 are known to be transmissible between humans or associated with human pandemics. However, subtypes H5, H6, H7, H9, and H10 can cross the species barrier from birds to mammals, including humans, and cause sporadic infections but have not yet acquired the ability to transmit between humans^5–8^. The reasons for this remain unclear. In 2013, three subtypes of AIVs were able to cross the species barrier and infect humans. In February 2013, an H7N9 AIV emerged in China and 1,566 laboratory-confirmed cases of human infection resulted in 613 deaths^9^. In May 2013, H6N1 AIV infections in humans occurred in Taiwan^10^. In December 2013, three human cases of H10N8 AIV infection were reported in China^5^.

A subtype H10 influenza virus was first isolated from a chicken in Germany in 1949^11^. Since then, H10 AIVs have been isolated with increasing frequency from wild and domestic aquatic and terrestrial avian species. H10 AIVs are now divided into two lineages: Eurasian and North American^12^. The first human infections with H10N7 AIV were reported in 2004 in Egypt^13^. An additional two cases were then confirmed in Australia in 2010^14^. Each of these human cases was associated with either interactions with, or proximity to, live poultry. H10N7 AIVs were isolated from hunted wild ducks in a live bird market in Egypt and from chickens processed on a farm in Australia that was associated with the 2010 H10N7 poultry outbreak. Those who acquired H10N8 infections were known to have a history of visiting live poultry markets (LPMs) or exposure to live poultry^5^. Currently, sustained human-to-human transmission has not been reported for H10 AIVs.

H10 AIVs have been isolated from various mammalian hosts, including harbor seals in northwestern Europe^15,16^. H10N8 AIV was infectious among feral dogs in LPMs in Guangdong Province, China^17^. Several Eurasian and North American–origin H10 AIVs replicate and cause weight loss in ferrets^18^. Therefore, H10 AIVs can cause disease in mammals and have the potential to pose a potential public health threat.

LPMs play a critical role in maintaining, amplifying, and disseminating AIVs among poultry species and from poultry to humans. After the first outbreak of highly pathogenic H5N1 AIV in 2007, ongoing active surveillance of poultry in Bangladesh has been conducted. A variety of AIV subtypes, including H5 and H9, have been isolated in LPMs in Bangladesh^19^. The circulation of H5N1 with other AIVs subtypes may increase the likelihood for emerging pandemic strains through reassortment. Reassortment between the polymerase basic 1 (PB1) gene from the H5N1 and H9N2 AIVs has been observed^20,21^. Recently, we described the emergence of a novel genotype H5N1 AIV containing the HA, M, and NA genes of circulating Bangladeshi H5N1 AIVs and five genes from low pathogenic Eurasian-lineage AIVs^22^.

During our active surveillance in Bangladesh, we isolated H10 AIVs from LPMs from 2008 to 2017. To better understand the evolution of these H10 AIVs, we sequenced their full genomes and analyzed their genetic and antigenic characteristics and receptor-binding properties. We determined the growth kinetics and antiviral susceptibility for these viruses and evaluated their pathogenic potential in mice.

## Results

### Isolation of AIVs from Bangladeshi LPMs

We collected 29,305 samples from LPMs through active surveillance in Bangladesh from 2008 to 2017. We screened all samples and confirmed the H5 subtype with quantitative reverse transcription PCR (qRT-PCR). We then subtyped all samples that tested negative for the H5 subtype by HA and NA sequencing. We detected eight different HA subtypes of AIVs, including H1 (5 isolates), H3 (12 isolates), H4 (4 isolates), H5 (182 isolates), H6 (3 isolates), H7 (1 isolate), H9 (1165 isolates), and H10 (8 isolates)^19^. We noted that eight H10 subtype AIVs were isolated from samples collected from three different LPMs in Bangladesh. All of the AIVs were isolated from ducks, with the exception of one H10N7 AIV that was isolated from a chicken (Table 1).

**Table 1:** H10 AIVs isolated from LPMs in Bangladesh.

Isolate	Subtype	Abbreviation	Host	GenBank Accession Numbers
A/duck/Bangladesh/821/2009	H10N7	DK/BD821/09 H10N7	Duck	MH071472, MH071458, MH071474, MH071475, MH071482, MH071500, MH071501, MH071464
A/duck/Bangladesh/822/2009	H10N7	DK/BD822/09 H10N7	Duck	MH071454, MH071477, MH071463, MH071479, MH071498, MH071481, MH071467, MH071499
A/duck/Bangladesh/824/2009	H10N7	DK/BD824/09 H10N7	Duck	MH071486, MH071502, MH071462, MH071452, MH071506, MH071496, MH071460, MH071473
A/chicken/Bangladesh/842/2009	H10N7	CK/BD842/09 H10N7	Chicken	MH071457, MH071493, MH071497, MH071465, MH071495, MH071470, MH071456, MH071491
A/duck/Bangladesh/8987/2010	H10N9	DK/BD8987/10 H10N9	Duck	MH071453, MH071461, MH071503, MH071490, MH071494, MH071484, MH071455, MH071488
A/duck/Bangladesh/8988/2010	H10N9	DK/BD8988/10 H10N9	Duck	MH071476, MH071492, MH071505, MH071468, MH071471, MH071478, MH071469, MH071483
A/duck/Bangladesh/24035/2014	H10N1	DK/BD24035/14 H10N1	Duck	KY616777, KY616773, KY616772, KY616787, KY616784, KY616756, KY616755, KY616753
A/duck/Bangladesh/24268/2015	H10N6	Dk/BD24268/15 H10N6	Duck	MH071485, MH071487, MH071451, MH071504, MH071459, MH071489, MH071480, MH071466

### Molecular and phylogenetic analysis of H10 AIVs

**Figure 1:**
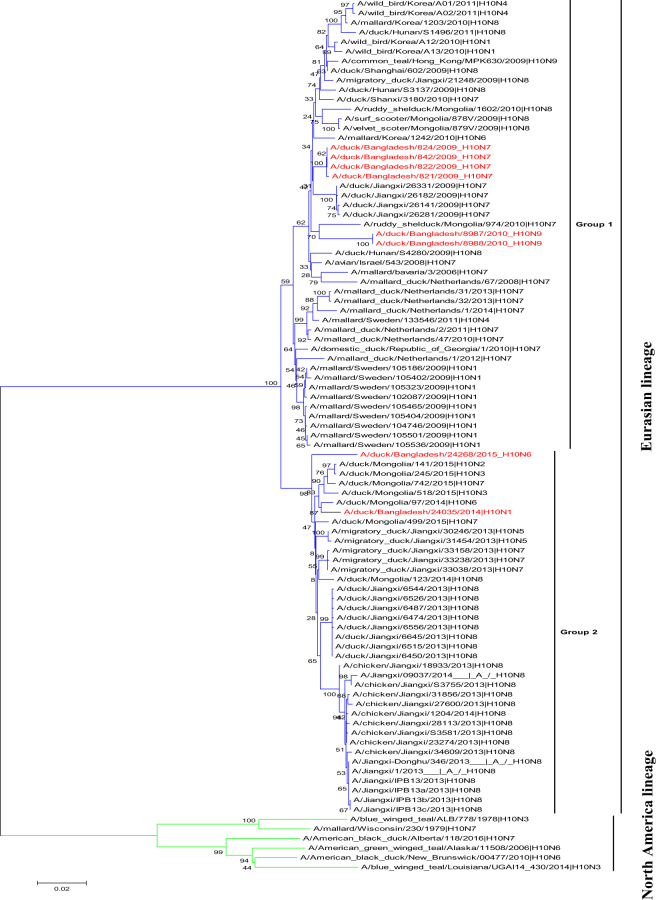
Phylogenetic trees for the HA gene of AIVs isolated from LPMs in Bangladesh. Phylogenetic analysis was performed with the neighbor-joining algorithm and the Kimura 2-parameter model. The reliability of the phylogenetic inference at each branch node was estimated by the bootstrap method with 1,000 replications. Evolutionary analyses were conducted with MEGA6 software. The H10 AIVs isolated during the surveillance period are denoted in red font.

To better understand the genetic relationship of the H10 viruses, we sequenced the complete genomes of eight H10 AIVs isolated from LPMs in Bangladesh. Phylogenetic analysis of the HA sequences revealed that they belonged to the Eurasian lineage. We classified the HA genes into two groups based on branch clustering of the phylogenetic tree (Fig. 1). Group 1 contained H10N7 and H10N9 AIVs in branches that also contained viruses from Europe and Asia. The HA gene of four H10N7 AIVs were most closely related to those of H10N7 AIVs isolated from Jiangxi, China in 2009. Group 2 contained H10N1 and H10N6 AIVs along with Asian AIVs and human H10N8 AIVs (Fig. 1).

The amino acid sequence of the HA protein of all H10 AIVs included a single arginine residue at the cleavage site of HA1 and HA2 (PELMQGR↓GLF), indicating that they were low pathogenic strains. We examined the HA proteins in these AIVs for the presence of N-linked glycosylation motifs by interrogating the proteins for N-(P-[S/T]-P) motifs, which indicate potential N-linked glycosylation sites. We identified four potential glycosylation sites in HA1 at positions 22, 38, 242, and 296, which are highly conserved in all H10 AIVs.

Phylogenetic analysis of the NA genes revealed that all NA subtypes (i.e., N1, N6, N7, and N9) belonged to the Eurasian lineage (Supplementary Fig. S1). The NA genes of four H10N7 AIVs were most closely related to those of H10N7 AIVs isolated from Jiangxi, China in 2009. The NA genes of the H10N9 and H10N6 AIVs were most closely related to those of AIVs isolated from free-range ducks in the Tanguar haor area in Bangladesh in 2015. This indicates that the NA genes may circulate in Bangladesh between poultry sold in LPMs and free-range ducks. The NA gene of the H10N1 AIV was most closely related to those of H6N1 AIVs that were previously isolated fromChina. In addition, we found that some North American–lineage AIVs from Alaska were related to those from the Eurasian lineage (Supplementary Fig. S1).

Phylogenetic analysis of the polymerase basic 2 (PB2) genes revealed that they formed two distinct groups within the Eurasian lineage, all belonging to the Eurasian lineage (Supplementary Fig. S2a). The PB2 gene of H10N7 and H10N9 AIVs were located in one group and shared 99% identity with the closest PB2 gene of A/ruddy shelduck/Mongolia/598C2/2009 (H7N3). The H10N6 AIV PB2 gene was most closely related to AIVs isolated from free-range ducks in the Tanguar haor area in Bangladesh. The PB2 gene of the H10N1 AIV shared 98% nucleotide identity with that of A/tufted duck/Mongolia/1409/2010 (H1N1).

The polymerase basic 1 (PB1) genes of eight H10 AIVs clustered into two groups and belonged to the Eurasian lineage (Supplementary Fig. S2b). All of our isolates expressed a PB1-F2 protein consisting of 90 amino acids. Furthermore, the H10N1 and H10N9 AIVs had an N66S substitution in PB1-F2 (Supplementary Table S1), which increases virulence and replication efficiency in mammalian hosts^23^. The polymerase acidic (PA) and nucleoprotein (NP) genes of the H10 AIVs clustered into two groups, all of which were of the Eurasian lineage (Supplementary Fig. S2c,d). The PA and NP genes of the H10N7 and H10N9 AIVs were related to those of AIVs isolated from Europe and Mongolia, whereas the NP gene of the H10N1 and H10N6 AIVs were most closely related to viruses isolated from Eastern China and free-range ducks in the Tanguar haor area in Bangladesh. The matrix (M) and nonstructural (NS) genes of all eight H10 AIVs segregated into two groups (Supplementary Fig. S2e,f). The amino acid substitutions at positions 26, 27, 30, 31, and 34 in the M2 protein facilitate resistance to some anti-influenza drugs (i.e., M2 ion channel blockers). We did not observe any of these substitutions in the H10 AIVs we isolated. However, we found that all H10 AIVs contained P42S and V149A substitutions in the NS1 protein, which are associated with virulence and pathogenicity in mammals^24,25^. We did not detect any deletions in the NS1 proteins in any of the H10 AIVs, and all were of allele A and Eurasian lineage.

### Antigenic analysis

To determine the antigenic relations among H10 AIVs isolated from LPMs in Bangladesh, we used a panel of polyclonal and monoclonal antibodies against Eurasian and North American viruses. The H10 AIVs were homogeneous and similar to those of the Eurasian lineage (Table 2). Four H10 AIVs reacted with antisera against A/laughing gull/Delaware Bay/209/2013 (H10N8) and A/glaucous gull/Iceland/CDX16-4552/2015 (H10N7) from North America, although hemagglutination inhibition (HI) assay titers with the H10N1 and H10N6 AIVs were one- to two-fold higher than were those of the other AIVs (Table 2). Two monoclonal antibodies against A/chicken/Jiangxi/34609/2013 (H10N8) cross-reacted with the H10 AIVs at higher titers and one monoclonal antibody to H10 influenza virus (JX13-3) failed to react with any of the Bangladeshi H10 viruses (Table 2). We found that the H10N7 and H10N9 AIVs from group 1, as classified by their HA gene phylogenetic trees, exhibited low cross-reactivity with antisera against group 2 AIVs (e.g., H10N1). These results indicate that the H10 AIVs circulating in LPMs in Bangladesh during the surveillance period were undergoing antigenic drift.

**Table 2:** Antigenic characterization by the hemagglutination inhibition assay of H10 AIVs isolated from LPMs in Bangladesh.

Virus	Monoclonal antibodies			Polyclonal antibodies*							
	JX13-3	JX13-4	JX13-5	N/49	HK-562	NL-1	NL-p14-221	JX13	DE-209	CDX16-4552	BD-24035
Reference virus											
A/chicken/Germany N/49 H10N7	<100	1600	<100	1280	80	160	80	320	320	160	80
A/duck/Hong Kong/562/79 H10N9	<100	3200	200	80	80	160	40	160	80	40	20
A/mallard/Netherlands/1/2014 H10N7	<100	3200	1600	80	160	160	80	160	80	80	20
A/chicken/Jiangxi/34609/2013 H10N8	3200	3200	400	1280	320	640	320	1280	640	320	80
Test virus											
A/duck/Bangladesh/821/2009 H10N7	<100	800	800	80	40	40	20	40	20	20	20
A/duck/Bangladesh/8988/2010 H10N9	<100	3200	400	80	160	160	80	160	80	40	40
A/duck/Bangladesh/24035/2014 H10N1	<100	3200	400	640	320	640	160	640	640	160	80
A/duck/Bangladesh/24268/2015 H10N6	<100	3200	400	320	320	320	160	320	320	160	80

*Polyclonal antibodies were produced in the ferret. Homologous titers are underlined. NL-p14-221, A/seal/Netherlands/p1/2014 (H10N7). DE-209, A/laughing gull/Delaware Bay/209/2013 (H10N8). CDX16-4552, A/glaucous gull/Iceland/CDX16-4552/2015 (H10N7).

### Replication of H10 AIVs in mice

**Figure 2:**
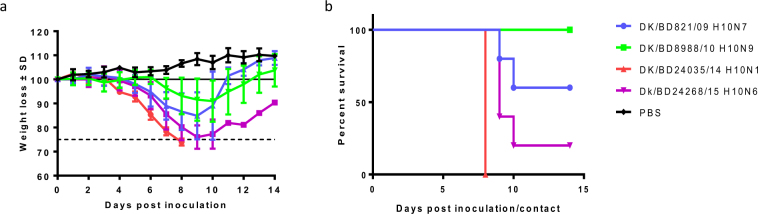
Morbidity and mortality of H10 AIVs in mice. DBA/2J mice (n = 5) were inoculated intranasally with 30 μL of 10^6^ EID_50_ DK/BD821/09 H10N7, DK/BD8988/10 H10N9, DK/BD24035/14 H10N1, or Dk/BD24268/15 H10N6. (**a**) Morbidity was examined by recording the body weights of inoculated mice daily, which is shown as the mean percent body weight from the day of inoculation (day 0). (**b**) Mortality was recorded as actual death or euthanasia at ≥25% weight loss, which was pre-established in our animal protocol.

To investigate the replication and virulence of H10 AIVs in mammals, we inoculated groups of 17 DBA/2J mice with 10^6^ 50% egg infective dose (EID50) of each AIV. Three mice in each group were euthanized on 2, 4, 6, and 8 days post inoculation (dpi) to determine viral titers in the nasal turbinate, lung, spleen, heart, intestine, and brain. We observed the remaining five mice in each group for 14 days to determine their susceptibility to these AIVs. Mice infected with DK/BD24035/14 (H10N1), Dk/BD24268/15 (H10N6), and DK/BD821/09 (H10N7) experienced significant weight loss (>25%) (Fig. 2a). Four viruses exhibited differing degrees of virulence in mice, The DK/BD24035/14 (H10N1) caused 100% mortality by 8 dpi; Dk/BD24268/15 (H10N6) caused 80% mortality by 10 dpi; DK/BD821/09 (H10N7) caused 40% mortality by day 10 dpi; and DK/BD8988/10 (H10N9) did not cause mortality (Fig. 2b).

**Figure 3:**
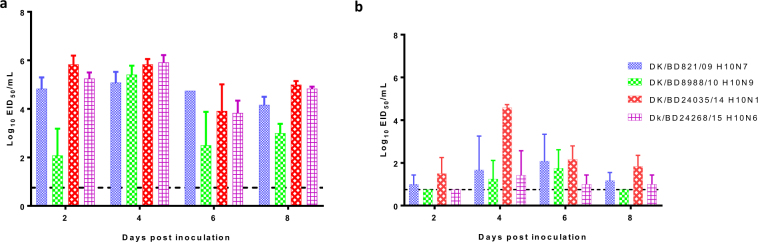
Replication of H10 AIVs in the lungs and nasal turbinates of inoculated mice. DBA/2J mice (n = 3) were infected with 10^6^ EID_50_ of DK/BD821/09 H10N7, DK/BD8988/10 H10N9, DK/BD24035/14 H10N1, or Dk/BD24268/15 H10N6. Lungs (**a**) and nasal turbinates (**b**) were collected at 2, 4, 6, and 8 dpi, and viral titers were determined by EID_50_ assays. The results are expressed as log_10_ EID_50_ of tissue; error bars depict standard deviations; and the dotted line indicates the lower limit of detection of infectious virus.

All viruses were detected in the lungs, with mean titers ranging from 3.1 to 5.8 log_10_ EID_50_ (Fig. 3a), and in nasal turbinates at all time points, with mean titers ranging from 1.5 to 4.75 log_10_ EID_50_ (Fig. 3b). The DK/BD24035/14 (H10N1) AIV titers were markedly higher in nasal turbinates than were the other isolates at 4 dpi (Fig. 3b). These data indicate that all the H10 isolates replicated in mice without prior adaptation.

We detected the two DK/BD24035/14 (H10N1) and Dk/BD24268/15 (H10N6) AIVs that caused high mortality in mice and were detected in the heart, liver, and brain tissues. In addition, we detected the DK/BD24035/14 (H10N1) AIV in the spleen at 2 and 4 dpi (Table 3). In contrast, we detected the DK/BD821/09 (H10N7) and DK/BD8988/10 (H10N9) AIVs in the heart and the DK/BD821/09 (H10N7) AIV in the liver at 2 and 4 dpi (Table 3). We did not detect any of the AIVs in the intestines of any of the mice.

**Table 3:** Replication kinetics of H10 AIVs in extrapulmonary tissues in mice.

Virus	Subtype	dpi	Viral titers (log_10_ EID_50_/mL)				
			Heart	Spleen	Liver	Intestine	Brain
A/duck/Bangladesh/821/2009	H10N7	2	2/3^a^ (2.5 ± 1.4)^b^	0/3	1/3 (2.5)	0/3	0/3
		4	1/3 (2.5)	0/3	1/3 (2.5)	0/3	0/3
		6	1/3 (2.5)	0/3	0/3	0/3	0/3
		8	2/3 (1.75 ± 0.35)	0/3	0/3	0/3	0/3
A/duck/Bangladesh/8988/2010	H10N9	2	0/3	0/3	0/3	0/3	0/3
		4	1/3 (3.25)	0/3	0/3	0/3	0/3
		6	1/3 (3.5)	0/3	0/3	0/3	0/3
		8	0/3	0/3	0/3	0/3	0/3
A/duck/Bangladesh/24035/2014	H10N1	2	2/3 (3 ± 0.35)	1/3 (1.5)	3/3 (2.92 ± 1.18)	0/3	3/3 (2.25 ± 0.43)
		4	1/3 (1.5)	1/3 (1.75)	0/3	0/3	1/3 (1.5)
		6	3/3 (2.08 ± 0.57)	0/3	2/3 (1.88 ± 0.53)	0/3	2/3 (1.88 ± 0.53)
		8	3/3 (1.92 ± 0.72)	0/3	2/3 (2.88 ± 1.94)	0/3	2/3 (2 ± 0.35)
A/duck/Bangladesh/24268/2015	H10N6	2	1/3 (1.5)	0/3	1/3 (3.25)	0/3	2/3 (2.13 ± 0.88)
		4	1/3 (1.5)	0/3	0/3	0/3	1/3 (2.75)
		6	1/3 (1.5)	0/3	0/3	0/3	1/3 (1.75)
		8	1/3 (1.5)	0/3	0/3	0/3	0/3

^a^Infected mice were positive for virus detection /Total infected mice.

^b^The titers are shown as the mean ± SD.

**Figure 4:**
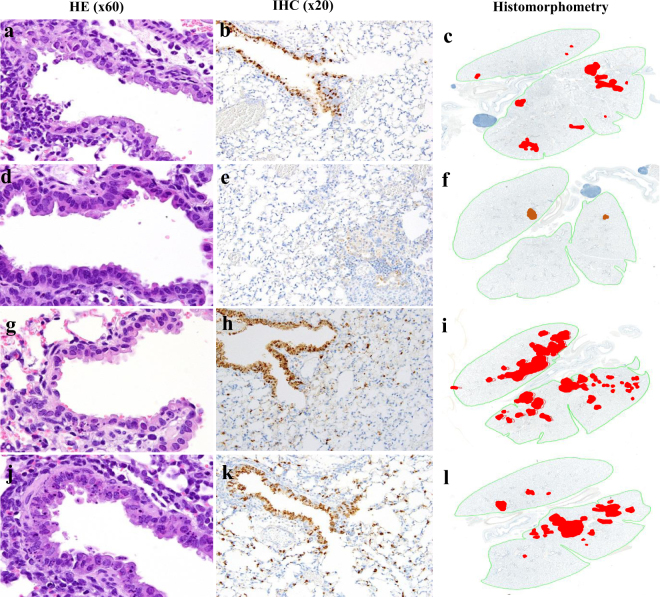
Pulmonary lesions and viral spread in the lungs of AIV-infected mice. Lungs infected with DK/BD821/09 H10N7 (**a**–**c**) showed scattered degenerating bronchiolar epithelial cells in terminal airways **(a)** but positive immunohistochemical (IHC) staining for viral antigen was mostly limited to bronchiolar epithelium **(b)** with only minimal spread to surrounding alveoli **(c)**. Following infections with DK/BD8988/10 H10N9 (**d**–**f**) there were no notable lesions in bronchiolar epithelium **(d)** and minimal viral antigen **(e)** was detected in the few inflammatory foci present **(f)**. In contrast, in lungs infected with either DK/BD24035/14 H10N1 (**g**–**i**) or Dk/BD24268/15 H10N6 (**j**–**l**), numerous apoptotic bronchiolar epithelial cells were evident in widely distributed terminal airways **(g**,**j)**, and IHC showed abundant virus-infected cells in both the terminal airways and extending into surrounding alveoli **(h**,**k)**. The virus-infection involved a much larger percentage of the total lung field **(I**,**l)** than the other two viruses (H10N7 and H10N9). Mouse lungs were fixed in 10% neutral buffered formalin and stained with hematoxylin and eosin (HE), subjected to IHC staining with anti–NP antiserum and analyzed by histomorphometry. Magnifications: 60× (HE), 20× (IHC), and 2× (histomorphometry). For histomorphometry, total lung areas are outlined in green, and areas with antigen-positive cells are shown in red.

Histopathologic analysis of the lungs of the mice infected with the DK/BD8988/10 (H10N9) AIV revealed minimal viral antigens in bronchiolar epithelial cells and none in alveolar cells. Infection with the DK/BD821/09 (H10N7) AIV caused intermittent infection of bronchiolar epithelial cells and minimal spread to alveoli. Both the DK/BD24035/14 (H10N1) and Dk/BD24268/15 (H10N6) AIVs caused diffuse infection of bronchiolar epithelial cells and spread to surrounding alveolar epithelial cells (Fig. 4).

### Replication of H10 AIVs in A549 cells

**Figure 5:**
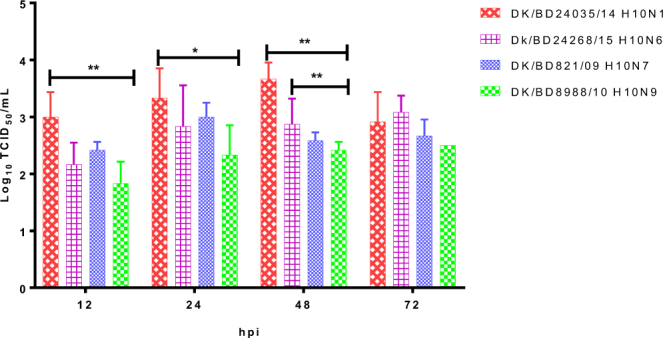
Growth characteristics of H10 AIVs in A549 cells. A549 cells were inoculated at a multiplicity of infection of 0.01 TCID_50_/cell with the DK/BD821/09 H10N7, DK/BD8988/10 H10N9, DK/BD24035/14 H10N1, or Dk/BD24268/15 H10N6 AIVs. Supernatants were collected at the indicated time points and titrated in MDCK cells by TCID_50_. Error bars depict standard deviations. *P < 0.05, **P < 0.01.

To determine the replication of H10 AIVs in human epithelial cells, we compared the multicycle growth kinetics of four viruses in the human A549 epithelial cell line. All four viruses grew efficiently in A549 cells; however, the viral titers of DK/BD24035/14 H10N1-infected cells were significantly higher than those of DK/BD8988/10 H10N9-infected cells at 12, 24, and 48 h post infection (P < 0.01 at 12 and 48 h post infection and P < 0.05 at 25 h, Fig. 5). The viral titers of DK/BD24035/14 H10N1-infected cells were also significantly higher than those of DK/BD821/09 H10N7-infected cells at 48 h postinfection (P < 0.01, Fig. 5).

### Receptor-binding assay

**Figure 6:**
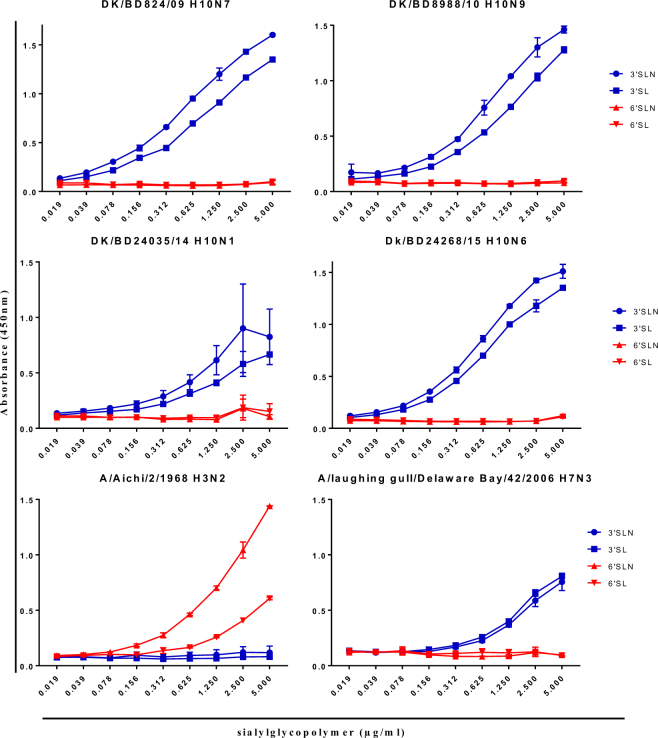
Receptor-binding specificity of H10 AIVs. Binding of H10 AIVs to the biotinylated sialylglycopolymers 3′ sialyllactosamine (3′SLN), 3′ sialyllactose (3′SL), 6′ sialyllactosamine (6′SLN), and 6′ sialyllactose (6′SL) were measured. A/Aichi/2/1968 (H3N2) and A/laughing gull/Delaware Bay/42/2006 (H7N3) were used as species-specific controls.

A switch in receptor-binding preference from avian-type receptors to human-type receptors is important for AIV transmission among humans. We tested the receptor-binding preferences of the H10 AIVs and found that all H10 AIVs had higher binding preferences for α2,3 avian-like receptors than for α2,6 human-like receptors (Fig. 6). Expectedly, we found the control A/Aichi/2/1968 (H3N2) human influenza A virus isolate bound to only the human-like receptor, whereas the avian derived virus A/laughing gull/Delaware Bay/42/2006 (H7N3) bound to only the avian-type receptor (Fig. 6).

### Antiviral susceptibility

To determine whether the Bangladeshi H10 AIVs were sensitive to currently approved antiviral drugs, we examined the susceptibility of these H10 AIVs to the NA inhibitors oseltamivir, zanamivir, and peramivir. All four H10 AIVs that we tested were fully susceptible to all three NA inhibitors, with mean 50% inhibitory concentration (IC_50_) values ranging from 0.12 to 3.06 nM (Table 4).

**Table 4:** Susceptibility of H10 AIVs from Bangladeshi LPMs to NA inhibitors.

Virus	Subtype	Inhibitory activity (IC_50_ ± SD, nM)*		
		Oseltamivir	Zanamivir	Peramivir
A/Mississippi/03/2001(WT)	H1N1	0.65 ± 0.06	0.28 ± 0.02	0.10 ± 0.01
A/Mississippi/03/2001(Res)	H1N1, H275Y	318.95 ± 22.00	0.32 ± 0.00	48.44 ± 0.97
B/Perth/211/2001 (WT)		22.96 ± 3.79	1.18 ± 0.05	0.37 ± 0.02
B/Perth/211/2001 (Res)	D187E	186.13 ± 39.83	5.25 ± 0.77	9.89 ± 0.96
A/duck/Bangladesh/821/2009	H10N7	1.10 ± 0.11	2.26 ± 0.32	0.54 ± 0.13
A/duck/Bangladesh/8988/2010	H10N9	0.76 ± 0.19	0.84 ± 0.10	0.14 ± 0.02
A/duck/Bangladesh/24035/2014	H10N1	3.06 ± 0.44	0.30 ± 0.03	0.12 ± 0.00
A/duck/Bangladesh/24268/2015	H10N6	0.74 ± 0.11	3.01 ± 0.58	0.43 ± 0.05

WT, wild-type virus; Res, control virus resistant to NA inhibitors.

*Inhibitory concentration (IC_50_) value is the concentration that inhibits viral NA activity by 50%. IC_50_ values are expressed as the mean ± SD (nM).

## Discussion

Fatal human cases of avian-origin H10N8 viral infections have caused increased interest in H10 subtype AIVs^5^. Extensive surveillance studies have identified H10 AIVs in waterfowl and LPMs^26–28^. Here, we isolated eight H10 AIVs from LPMs in Bangladesh through active surveillance from 2008 to 2017. Our findings demonstrate that these isolates belong to the Eurasian lineage and replicate efficiently in the respiratory system of mice, without adaptation and with some spread beyond the respiratory tract. This enhanced virulence in mice highlights the need for continued monitoring of AIVs in LPMs, and evaluation of the resultant isolates to identify and understand the pathogenic mechanisms of emerging strains with the potential to infect and cause disease in humans.

Our phylogenetic analysis of the H10 HA gene revealed that two different groups of Eurasian H10 AIVs were introduced into Bangladesh. The group 2 AIVs H10N1 and H10N6 were similar to H10N8 AIVs that formed a stable lineage in Jiangxi Province, China^26^, suggesting that H10 AIVs may have reassorted with other subtypes in Bangladesh. Our phylogenetic analysis of the NA gene of the H10N1 AIV revealed branching with the NA genes detected in wild and domestic ducks in China. In contrast, the NA genes of H10N6 AIVs branched with those from AIVs recently detected in the Tanguar haor area of Bangladesh and showed similarities to those of Mongolian AIVs. The NA and HA genes of the H10N7 AIVs were similar to those of H10N7 from Jiangxi, China. This suggests that some viruses were introduced to LPMs and others reassorted with viruses circulating in the LPMs or free-range ducks in Bangladesh. The internal genes of the H10 AIVs were more complex and shared high homology with other subtypes of AIVs. Therefore, epidemiologic monitoring of AIVs in migratory birds is essential to better understand their transmission, reassortment and evolution. Antigenic analysis of HA of the H10 AIVs indicated that they share common epitopes. We detected antigenic differences between the A/chicken/Jiangxi/34609/2013 (H10N8) AIV and the other H10 AIVs that correlated with substitutions in the HA at residues 137 and 83 at antigenic sites A and E, respectively. Overall, we observed limited antigenic drift among the North American and Eurasian lineages.

Migratory birds are important in the evolution, maintenance, and spread of AIVs. Bangladesh is located in the Central Asian flyway and is near the Eastern Asian-Australian and Black Sea-Mediterranean flyways, and is a stopover site for migratory birds. The interaction between wild birds and domestic and/or free-range ducks, which introduces AIVs into poultry systems, was responsible for emerging new strains that infected humans. This was observed in the emergence of novel H7N9 and H10N8 strains in China in 2013^28,29^. We previously showed that the viruses isolated from Tanguar haor area played a role in the development of a new genotype of H5N1 clade 2.3.2.1a through reassortment^22^. Therefore, further study is needed to address the link between AIVs isolated from free-range ducks in the Tanguar haor area and the diversity and evolution of AIVs circulating in LPMs in Bangladesh.

The mouse model has been widely used to evaluate the virulence of AIVs in mammals. We used DBA/2J mice to evaluate the disease potential of H10 AIVs because DBA/2J mice are more sensitive to influenza A infections than are other strains^30–32^. We found that all H10 AIVs infected and replicated in mice without prior adaptation, some of the AIVs caused some degree of weight loss, and some were lethal. Furthermore, these AIVs replicated to higher titers in the lungs than in the nasal turbinates, which is consistent with previous findings showing that H10N8 AIVs isolated from LPMs in China replicated well in the respiratory tracts of mice, with higher viral loads in the lungs than in nasal turbinates^33^.

Mouse studies can be useful to investigate the potential of low pathogenic AIVs to replicate and cause disease and also can be used to discover the molecular markers which are important for adaptation to a mammalian host^34^. Recent studies demonstrated that many low pathogenic H1 and H7 AIVs isolated from wild birds in North America caused mortality in mice prior to adaptation^31,35^. Recently, we described the replication and pathogenic potential of H3, H7, and H15 viruses from free-range ducks in Bangladesh using mice. We observed that H7N1 and H7N9 viruses caused 100% and 60% mortality respectively, and expanded their tissue tropism beyond the pulmonary tissues to heart, liver, and brain^36^. Our current study showed this for both H10N1 and H10N6 AIVs as well as high mortality in mice.

The PB2 E627K substitution has been shown to confer increased virulence of AIVs in mammals^37,38^. All of the H10 AIVs we isolated did not contain the PB2 E627K substitution. The E627K substitution is present in viruses obtained from human samples infected with H10N8 AIVs but not in poultry isolates^26,27^, suggesting that this substitution is required to improve replication in mammalian hosts. The NS gene of H10N4 AIV has also been shown to contribute to virulence^39^. In 2014, an H10N7 AIV was isolated from seals in northeastern Europe that caused severe mortality^40,41^. An H10N4 AIV was also detected in mink farms in Sweden^42^. We observed that all H10 AIVs had mutations in their NS1 genes that resulted in a P42S substitution, which is associated with virulence and pathogenicity in mammals^24,25^.

AIV mutations conferring resistance to oseltamivir emerged after the antiviral was used to treat patients with H7N9 and H10N8 AIV infections^26,43^. All of the H10 AIVs we tested in this study were susceptible to NA inhibitors. However, monitoring influenza viruses for the presence of drug-resistance markers is important to predict the emergence of strains with such markers.

The H9N2 AIV is endemic in Bangladesh^21,44–46^ and has acquired mammalian host–specific mutations in its internal genes, which have been shown to facilitate transmission from avian species to humans^44^. H9N2 AIVs are significant donors of genetic material to emerging zoonotic viruses such as H5Nx, H7N9, and H10N8 AIVs posing an enormous threat to both human health and poultry industry. The wide circulation of H9N2 AIVs in Bangladeshi LPMs affords H9N2 with more opportunities for reassortment with other AIV subtypes, such as H10 AIVs. Transmission of H10 AIVs to humans has resulted in some fatal cases, and serologic evidence indicates that H10 AIVs were previously transmitted among turkey farmers in the United States^47^. This should raise concern about the potential for human-to-human transmission of H10 AIVs. Evaluation of the biologic properties of H10 and other subtypes of AIVs circulating in LPMs is essential for understanding the emergence and evolution of these viruses and to reduce their potential pandemic threat to public health.

## Materials and Methods

### Ethics statement

All mouse experiments were approved and performed according to the guidelines set by the Animal Care and Use Committee of St. Jude Children’s Research Hospital in an enhanced Animal Biosafety Level 3 containment facility.

### Virus isolation

The H10 AIVs used in this study were isolated from LPMs between 2008 and 2017 in Bangladesh during routine surveillance. The HA and NA subtypes of the AIVs were identified via qRT-PCR. All viruses were propagated and titrated for EID_50_ in the allantoic cavities of 10-day-old embryonated chicken eggs at 35 °C for 48 h. Virus titers were determined by injecting 100 μL of serial 10-fold dilutions of virus into the allantoic cavities of 10-day-old embryonated chicken eggs and then calculating the EID_50_ according to the method of Reed and Muench^48^.

### Deep amplicon sequencing and genetic analysis

Viral RNA was extracted by using an RNeasy kit (QIAGEN); conventional RT-PCR was then performed by using a SuperScript III first-strand synthesis kit (Invitrogen) with the Uni12 influenza primer. Multiplex PCR of all eight gene segments was conducted by using PCR Supermix HiFi (Invitrogen) with the Uni12/13 primers. PCR products were purified on a spin column (QIAGEN). DNA libraries were prepared by using NEXTera XT DNA-Seq library prep kits (Illumina) with 96 dual-index barcodes, according to manufacturer instructions. Pooled libraries were sequenced with an Illumina MiSeq Personal Genome Sequencer and 150 bp paired-end reads. The closest relatives of the viral genes sequenced of the eight isolated viruses were identified in the Global Initiative on Sharing Avian Influenza Data (GISAID) database and the Influenza Sequence Database (ISD)^49,50^. For phylogenetic analysis, multiple-sequence alignments for each data set were conducted using the MAFFT program^51^. MEGA6 was used to construct phylogenetic trees by applying the neighbor-joining method with the Kimura two-parameter distance model and 1,000 bootstrap replicates^52^. Several virus sequences of the North American lineage were also used as an out group in phylogenetic trees.

### Antigenic characterization

The HI assay was used to antigenically characterize the viruses. The four H10 AIVs were tested by using reference antisera against H10 AIVs selected from the North American and Eurasian lineages. The antisera against DK/BD24035/14 (H10N1) was generated for this study. Briefly, ferrets were intranasally infected with 1 mL of 10^6^ EID_50_/mL viruses and then boosted after 3 weeks by intramuscular injection of virus with adjuvant. Blood was collected 1 week later for serum isolation. A panel of three mouse monoclonal antibodies (JX13-3, JX13-4, and JX13-4) prepared against the H10 HA of A/chicken/Jiangxi/34609/2013 (H10N8) virus. Monoclonal antibodies were prepared at St. Jude Children’s Research Hospital by ClonaCell-HY Hybridoma Kit (StemCell Technologies) according to manufacturer instructions. Anti-H10 monoclonal antibodies were submitted to BEI Research Resources https://www.beiresources.org/ under catalog no. NR-50408, NR-50409, and NR-50410. The HI test was performed according to WHO protocols^53^.

### Pathogenicity in mice

Four H10 isolates were selected to investigate H10 AIVs pathogenicity in DBA/2J mice. Groups of seventeen mice were anesthetized with isoflurane and intranasally inoculated with 30 µL of 10^6^ EID_50_/mL virus, and an uninfected group was anesthetized and intranasally inoculated with 30 µL of phosphate-buffered saline, pH 7.2 (PBS) as controls. Upon virus challenge, five mice per group were monitored for 14 dpi for disease signs, weight loss, and mortality. Mortality was recorded as actual death or loss of ≥25% body weight, which is the threshold in which animals are required to be euthanized according to our animal protocol. Three mice per group were euthanized via CO_2_ asphyxiation at 2, 4, 6, and 8 dpi. Lung, intestine, liver, brain, spleen, and heart were collected to determine viral titers. Whole organs of lung, liver, brain, spleen, heart, and parts from intestine were homogenized in 1 ml PBS containing antibiotics with a Qiagen Tissue Lyser II (Qiagen, Gaithersburg, MD). Organ homogenates were centrifuged at 2000*g* for 10 min, and the supernatants were transferred to clean tubes from which 10-fold dilutions (10^−1^ to 10^−10^) were made in a solution of sterile PBS and antibiotics. For each dilution, three 10- to 11-day-old chicken embryos were inoculated via the allantoic cavity to determine the viral titers for each organ in terms of EID_50_.

### Histology and immunohistochemistry

The lungs of mice (n = 3) were collected at 3 dpi and fixed via intratracheal infusion and immersion in a 10% neutral buffered formalin solution. Tissues were embedded in paraffin, sectioned, and stained with hematoxylin and eosin. Immunohistochemical staining was performed with serial histologic sections to determine the distribution of AIV antigens. A goat primary polyclonal antibody (US Biological, Swampscott, MA) against anti-influenza A, USSR (H1N1) was used (1:1,000) on tissue sections that were previously subjected to antigen retrieval for 30 min at 98 °C (Target Retrieval Solution, pH 9; Dako Corp., Carpinteria, CA). To quantify the extent of viral infection in the lungs, digital images of whole lung sections stained for viral antigens were acquired with an Aperio ScanScope XT Slide Scanner (Leica Biosystems, Buffalo Grove, IL). Both uninfected and virus-positive regions were then manually outlined, and the areas of the outlined regions were determined with ImageScope software (Leica Biosystems).

### Viral replication in human lung cells

Monolayers of A549 cells were inoculated at a multiplicity of infection of 0.01 50% tissue culture infectious dose (TCID_50_) with DK/BD821/09 (H10N7), DK/BD8988/10 (H10N9), DK/BD24035/14 (H10N1), and Dk/BD24268/15 (H10N6). Cells were incubated at 37 °C in RPMI containing 0.2 µg/mL of L-1-tosylamido-2-phenylethyl chloromethyl ketone–treated trypsin (Sigma-Aldrich, St. Louis, MO). Supernatants were collected at different time points and titrated in MDCK cells by TCID_50_.

### Receptor specificity assay

Receptor specificity of H10 AIVs was analyzed by using a solid-phase direct-binding assay, as described previously^54^. Four different glycopolymers were used: biotinylated glycans α2,3 [Neu5Acα2-3Galβ1-4GlcNAcβ-PAA-biotin (3′SLN) and Neu5Acα2-3Galβ 1,4Glc β-PAA-biotin (3′SL)] and α2,6 [Neu5Aca2-6Galβ1-4Glcβ-PAA-biotin (6′SLN) and Neu5Acα2-3Galβ 1,4Glc β-PAA-biotin(6′SL)]. A/Aichi/2/1968 (H3N2) was used as a specificity control for α2,6 binding and A/laughing gull/Delaware Bay/42/2006 (H7N3) was used as a specificity control for α2,3 binding. Binding was detected by measuring absorbance at 450 nm.

### Susceptibility of H10 AIVs to NA inhibitors

The NA inhibitors oseltamivir carboxylate (oseltamivir; Hoffmann-La Roche, Basel, Switzerland), zanamivir (GlaxoSmithKline, Research Triangle Park, NC, USA), and peramivir (BioCryst Pharmaceuticals, Birmingham, AL, USA) were used to test the sensitivity of the H10 AIVs to NA inhibitors. A fluorescence-based NA inhibition assay with the fluorogenic substrate 2′-(4-methylumbelliferyl)-α-D-N-acetylneuraminic acid (MUNANA; Sigma-Aldrich, St Louis, MO) was used to determine IC_50_ values^55,56^.

### Statistical analysis

All statistical analyses were performed by two-way ANOVA in combination with Bonferroni multiple comparison tests by using GraphPad Prism 5.0 (GraphPad Software, La Jolla, CA). P values < 0.05 were considered statistically significant.

### Nucleotide sequence accession numbers

The nucleotide sequences of the eight H10 AIVs described in this study were deposited in the GenBank database with the accession numbers shown in Table 1.

## Electronic supplementary material

Supplementary Information
