# Supplementary material for: Genetic characterization and pathogenic potential of H10 avian influenza viruses isolated from live poultry markets in Bangladesh
Source: Sci Rep. 2018 Jul 16;8:10693. doi: 10.1038/s41598-018-29079-1 (PMC6048039; doi:10.1038/s41598-018-29079-1)
Supplement: Supplementary file 1 — Supplementary Information [file 41598_2018_29079_MOESM1_ESM.pdf]

Genetic characterization and pathogenic potential of H10 avian influenza viruses isolated from live poultry markets in Bangladesh

Rabeh El-Shesheny[1,2], John Franks[1], Bindumadhav M. Marathe[1], M. Kamrul Hasan[3], Mohammed M. Feeroz[3], Scott Krauss[1], Peter Vogel[4], Pamela McKenzie[1], Richard J. Webby[1] & Robert G. Webster[1]

[1]Department of Infectious Diseases, St. Jude Children's Research Hospital, Memphis, TN 38105, USA
[2]Center of Scientific Excellence for Influenza Viruses, National Research Centre, Giza, Egypt
[3]Department of Zoology, Jahangirnagar University, Dhaka 1342, Bangladesh
[4]Department of Pathology, St Jude Children's Research Hospital, Memphis, TN 38105, USA

Correspondence: Robert G. Webster, Department of Infectious Diseases, MS 330, St. Jude Children's Research Hospital, 262 Danny Thomas Place, Memphis, TN 38105-3678, USA; tel.: 901-595-3400; fax: 901-595-8559; email: robert.webster@stjude.org

Supplement Figure Legends

Figure S1. Phylogenetic analyses of the NA genes of AIVs isolated from LPMs in Bangladesh. Phylogenetic analysis was performed with the neighbor-joining algorithm and the Kimura 2-parameter model. The reliability of the phylogenetic inference at each branch node was estimated by the bootstrap method with 1,000 replications. Evolutionary analyses were conducted with MEGA6 software. The H10 AIVs isolated during the surveillance period are denoted in red font. Viruses isolated from Tanguar haor are denoted in blue font.

Figure S2. Phylogenetic analyses of the PB2, PB1, PA, NP, M, and NS genes of H10 AIVs. Phylogenetic analyses of the PB2 (**a**), PB1 (**b**), PA (**c**), NP (**d**), M (**e**), and NS (**f**) genes were performed with the neighbor-joining algorithm and the Kimura 2-parameter model. The reliability of the phylogenetic inference at each branch node was estimated by the bootstrap method with 1,000 replications. Evolutionary analyses were conducted with MEGA6 software. The H10 AIVs isolated during the surveillance period are denoted in red font. Viruses isolated from Tanguar haor are denoted in blue font.

Virus	HA														PB2							PB1			PA		M2	NS1	
	Cleavage site	RBS					antigenic site A			B			C	E	E627K	**L89V**	G309D	T339K	R477G	I495V	D701N	L13P	V336I	I368V	K356R	T409S	S31N	P42S	V149A
		E190D	G225D	Q226L	S138A	G228S	137	144	145	156	158	193	53	83															
A/Jiangxi-Donghu/346/2013 H10N8	PELIQGR↓GLF	E	G	Q	A	G	R	G	N	S	G	D	M	M	K	V	D	K	G	V	D	P	V	V	R	N	N	S	A
A/duck/Bangladesh/821/2009 H10N7	PEIMQGR↓GLF	E	G	Q	A	G	K	G	N	S	G	N	M	T	E	V	D	K	G	V	D	P	V	I	K	S	S	S	A
A/duck/Bangladesh/822/2009 H10N7	PEIMQGR↓GLF	E	G	Q	A	G	K	G	N	S	G	N	M	T	E	V	D	K	G	V	D	P	I	I	K	S	S	S	A
A/duck/Bangladesh/824/2009 H10N7	PEIMQGR↓GLF	E	G	Q	A	G	K	G	N	S	G	N	M	T	E	V	D	K	G	V	D	P	V	I	K	S	S	S	A
A/duck/Bangladesh/842/2009 H10N7	PEIMQGR↓GLF	E	G	Q	A	G	K	G	N	S	G	N	M	T	E	V	D	K	G	V	D	P	V	I	K	S	S	S	A
A/duck/Bangladesh/8987/2010 H10N9	PEIMQGR↓GLF	E	G	Q	A	G	K	G	N	S	G	D	V	T	E	V	D	K	G	V	D	P	V	I	K	S	S	S	A
A/duck/Bangladesh/8988/2010 H10N9	PEIMQGR↓GLF	E	G	Q	A	G	K	G	N	S	G	D	V	T	E	V	D	K	G	V	D	P	V	I	K	S	S	S	A
A/duck/Bangladesh/24035/2014 H10N1	PELMQGR↓GLF	E	G	Q	A	G	K	G	N	S	G	D	M	T	E	V	D	K	G	V	D	P	V	I	K	S	S	S	A
A/duck/Bangladesh/24268/2015 H10N6	PELMQGR↓GLF	E	G	Q	A	G	K	G	N	S	G	D	M	T	E	V	D	K	G	A	D	P	V	I	K	S	S	S	A

Table S1. **Comparison of amino acid sequences of H10 AIVs isolated from LPMs in Bangladesh.**

Fig. S1a

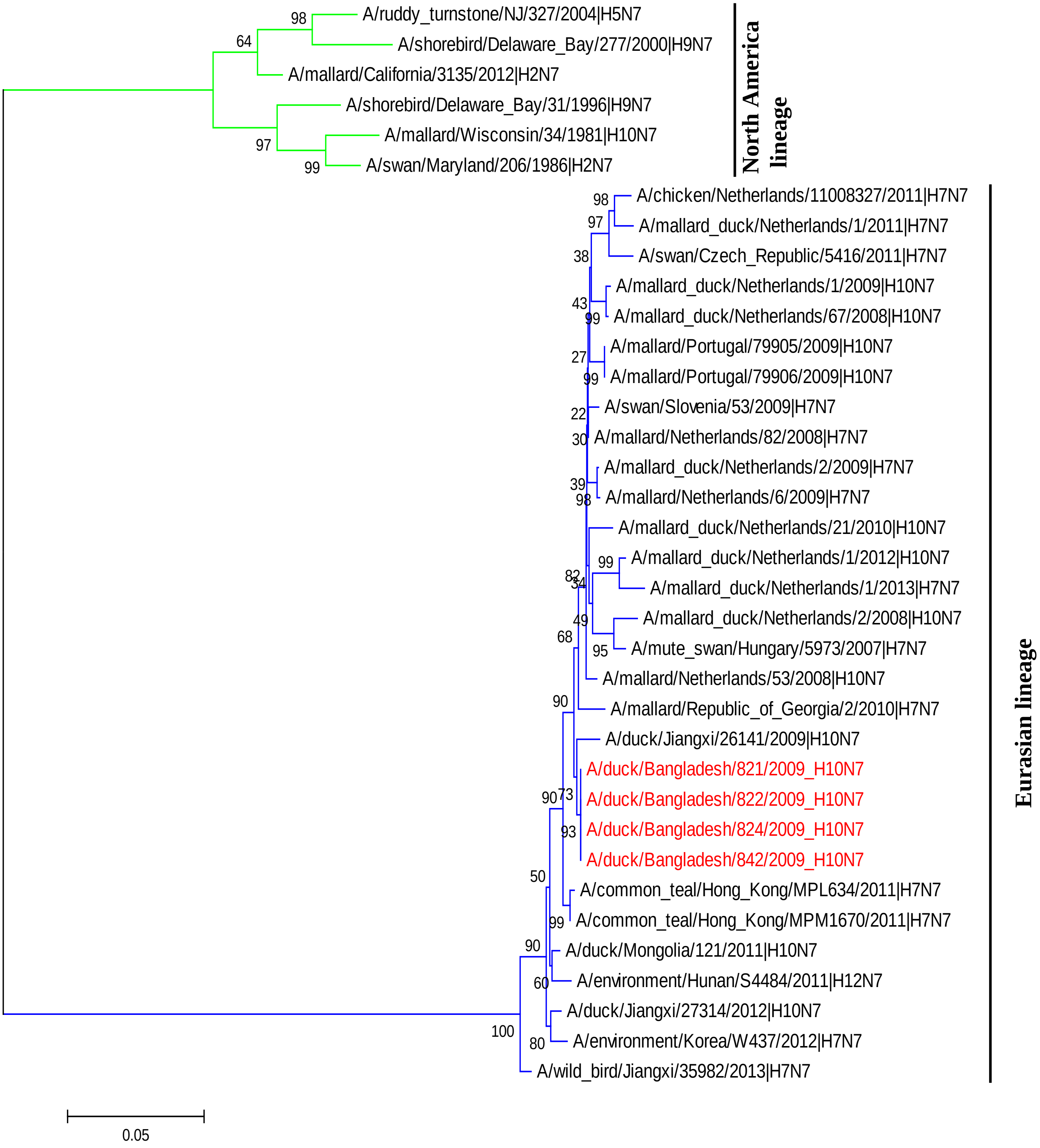
A/ruddy_turnstone/NJ/327/2004|H5N7
A/shorebird/Delaware_Bay/277/2000|H9N7
A/mallard/California/3135/2012|H2N7
A/shorebird/Delaware_Bay/31/1996|H9N7
A/mallard/Wisconsin/34/1981|H10N7
A/swan/Maryland/206/1986|H2N7
North America lineage
A/chicken/Netherlands/11008327/2011|H7N7
A/mallard_duck/Netherlands/1/2011|H7N7
A/swan/Czech_Republic/5416/2011|H7N7
A/mallard_duck/Netherlands/1/2009|H10N7
A/mallard_duck/Netherlands/67/2008|H10N7
A/mallard/Portugal/79905/2009|H10N7
A/mallard/Portugal/79906/2009|H10N7
A/swan/Slovenia/53/2009|H7N7
A/mallard/Netherlands/82/2008|H7N7
A/mallard_duck/Netherlands/2/2009|H7N7
A/mallard/Netherlands/6/2009|H7N7
A/mallard_duck/Netherlands/21/2010|H10N7
A/mallard_duck/Netherlands/1/2012|H10N7
A/mallard_duck/Netherlands/1/2013|H7N7
A/mallard_duck/Netherlands/2/2008|H10N7
A/mute_swan/Hungary/5973/2007|H7N7
A/mallard/Netherlands/53/2008|H10N7
A/mallard/Republic_of_Georgia/2/2010|H7N7
A/duck/Jiangxi/26141/2009|H10N7
A/duck/Bangladesh/821/2009_H10N7
A/duck/Bangladesh/822/2009_H10N7
A/duck/Bangladesh/824/2009_H10N7
A/duck/Bangladesh/842/2009_H10N7
A/common_teal/Hong_Kong/MPL634/2011|H7N7
A/common_teal/Hong_Kong/MPM1670/2011|H7N7
A/duck/Mongolia/121/2011|H10N7
A/environment/Hunan/S4484/2011|H12N7
A/duck/Jiangxi/27314/2012|H10N7
A/environment/Korea/W437/2012|H7N7
A/wild_bird/Jiangxi/35982/2013|H7N7
Eurasian lineage
0.05

Fig. S1b

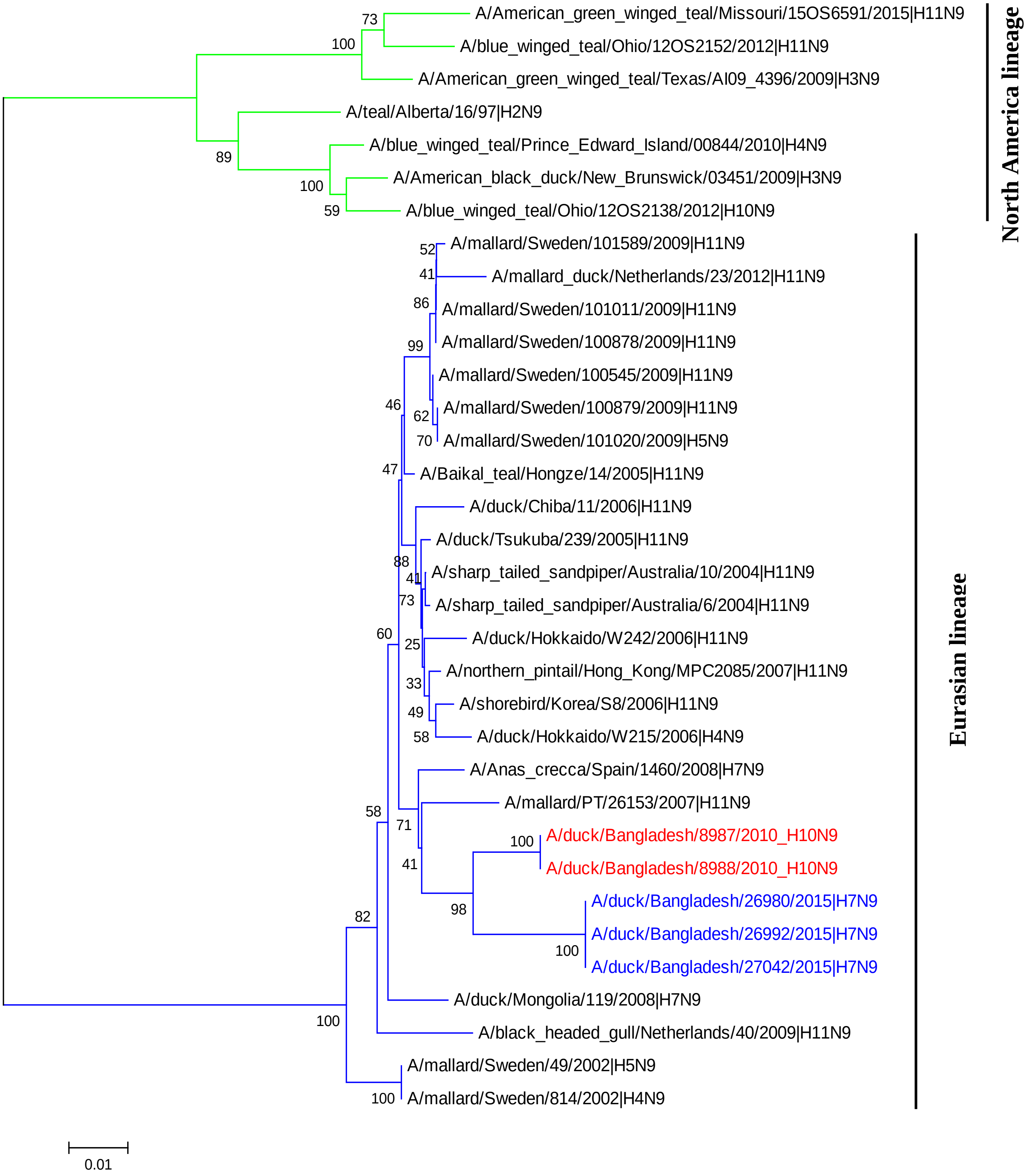

A/American_green_winged_teal/Missouri/15OS6591/2015|H11N9
A/blue_winged_teal/Ohio/12OS2152/2012|H11N9
A/American_green_winged_teal/Texas/AI09_4396/2009|H3N9
A/teal/Alberta/16/97|H2N9
A/blue_winged_teal/Prince_Edward_Island/00844/2010|H4N9
A/American_black_duck/New_Brunswick/03451/2009|H3N9
A/blue_winged_teal/Ohio/12OS2138/2012|H10N9
A/mallard/Sweden/101589/2009|H11N9
A/mallard_duck/Netherlands/23/2012|H11N9
A/mallard/Sweden/101011/2009|H11N9
A/mallard/Sweden/100878/2009|H11N9
A/mallard/Sweden/100545/2009|H11N9
A/mallard/Sweden/100879/2009|H11N9
A/mallard/Sweden/101020/2009|H5N9
A/Baikal_teal/Hongze/14/2005|H11N9
A/duck/Chiba/11/2006|H11N9
A/duck/Tsukuba/239/2005|H11N9
A/sharp_tailed_sandpiper/Australia/10/2004|H11N9
A/sharp_tailed_sandpiper/Australia/6/2004|H11N9
A/duck/Hokkaido/W242/2006|H11N9
A/northern_pintail/Hong_Kong/MPC2085/2007|H11N9
A/shorebird/Korea/S8/2006|H11N9
A/duck/Hokkaido/W215/2006|H4N9
A/Anas_crecca/Spain/1460/2008|H7N9
A/mallard/PT/26153/2007|H11N9
A/duck/Bangladesh/8987/2010_H10N9
A/duck/Bangladesh/8988/2010_H10N9
A/duck/Bangladesh/26980/2015|H7N9
A/duck/Bangladesh/26992/2015|H7N9
A/duck/Bangladesh/27042/2015|H7N9
A/duck/Mongolia/119/2008|H7N9
A/black_headed_gull/Netherlands/40/2009|H11N9
A/mallard/Sweden/49/2002|H5N9
A/mallard/Sweden/814/2002|H4N9
73
100
89
100
59
52
41
86
99
46
62
70
47
88
41
73
60
25
33
49
58
58
71
100
41
98
82
100
100
100
North America lineage
Eurasian lineage
0.01

Fig. S1c

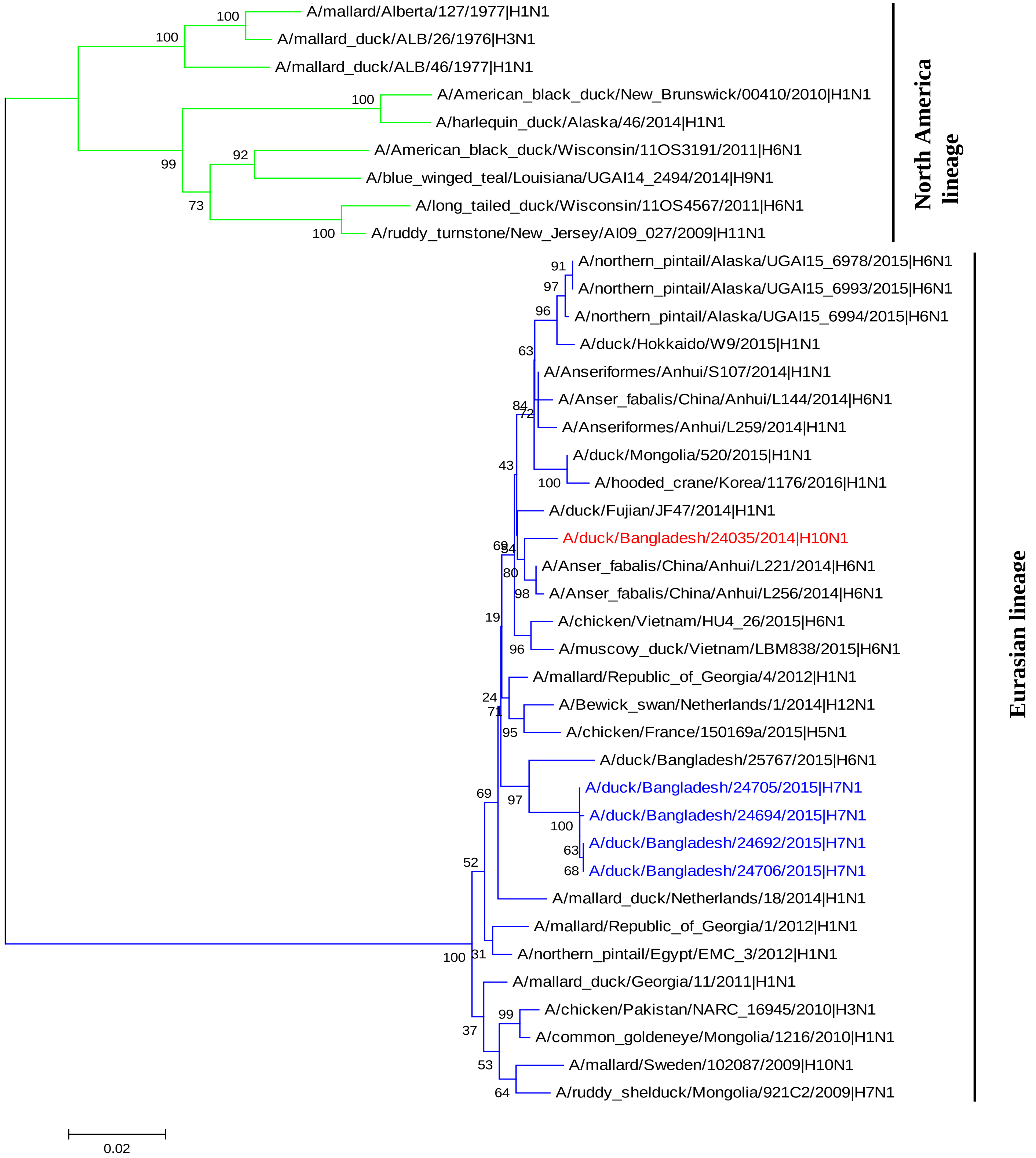

A/mallard/Alberta/127/1977|H1N1
A/mallard_duck/ALB/26/1976|H3N1
A/mallard_duck/ALB/46/1977|H1N1
A/American_black_duck/New_Brunswick/00410/2010|H1N1
A/harlequin_duck/Alaska/46/2014|H1N1
A/American_black_duck/Wisconsin/11OS3191/2011|H6N1
A/blue_winged_teal/Louisiana/UGAI14_2494/2014|H9N1
A/long_tailed_duck/Wisconsin/11OS4567/2011|H6N1
A/ruddy_turnstone/New_Jersey/AI09_027/2009|H11N1
North America lineage
A/northern_pintail/Alaska/UGAI15_6978/2015|H6N1
A/northern_pintail/Alaska/UGAI15_6993/2015|H6N1
A/northern_pintail/Alaska/UGAI15_6994/2015|H6N1
A/duck/Hokkaido/W9/2015|H1N1
A/Anseriformes/Anhui/S107/2014|H1N1
A/Anser_fabalis/China/Anhui/L144/2014|H6N1
A/Anseriformes/Anhui/L259/2014|H1N1
A/duck/Mongolia/520/2015|H1N1
A/hooded_crane/Korea/1176/2016|H1N1
A/duck/Fujian/JF47/2014|H1N1
A/duck/Bangladesh/24035/2014|H10N1
A/Anser_fabalis/China/Anhui/L221/2014|H6N1
A/Anser_fabalis/China/Anhui/L256/2014|H6N1
A/chicken/Vietnam/HU4_26/2015|H6N1
A/muscovy_duck/Vietnam/LBM838/2015|H6N1
A/mallard/Republic_of_Georgia/4/2012|H1N1
A/Bewick_swan/Netherlands/1/2014|H12N1
A/chicken/France/150169a/2015|H5N1
A/duck/Bangladesh/25767/2015|H6N1
A/duck/Bangladesh/24705/2015|H7N1
A/duck/Bangladesh/24694/2015|H7N1
A/duck/Bangladesh/24692/2015|H7N1
A/duck/Bangladesh/24706/2015|H7N1
A/mallard_duck/Netherlands/18/2014|H1N1
A/mallard/Republic_of_Georgia/1/2012|H1N1
A/northern_pintail/Egypt/EMC_3/2012|H1N1
A/mallard_duck/Georgia/11/2011|H1N1
A/chicken/Pakistan/NARC_16945/2010|H3N1
A/common_goldeneye/Mongolia/1216/2010|H1N1
A/mallard/Sweden/102087/2009|H10N1
A/ruddy_shelduck/Mongolia/921C2/2009|H7N1
Eurasian lineage
0.02

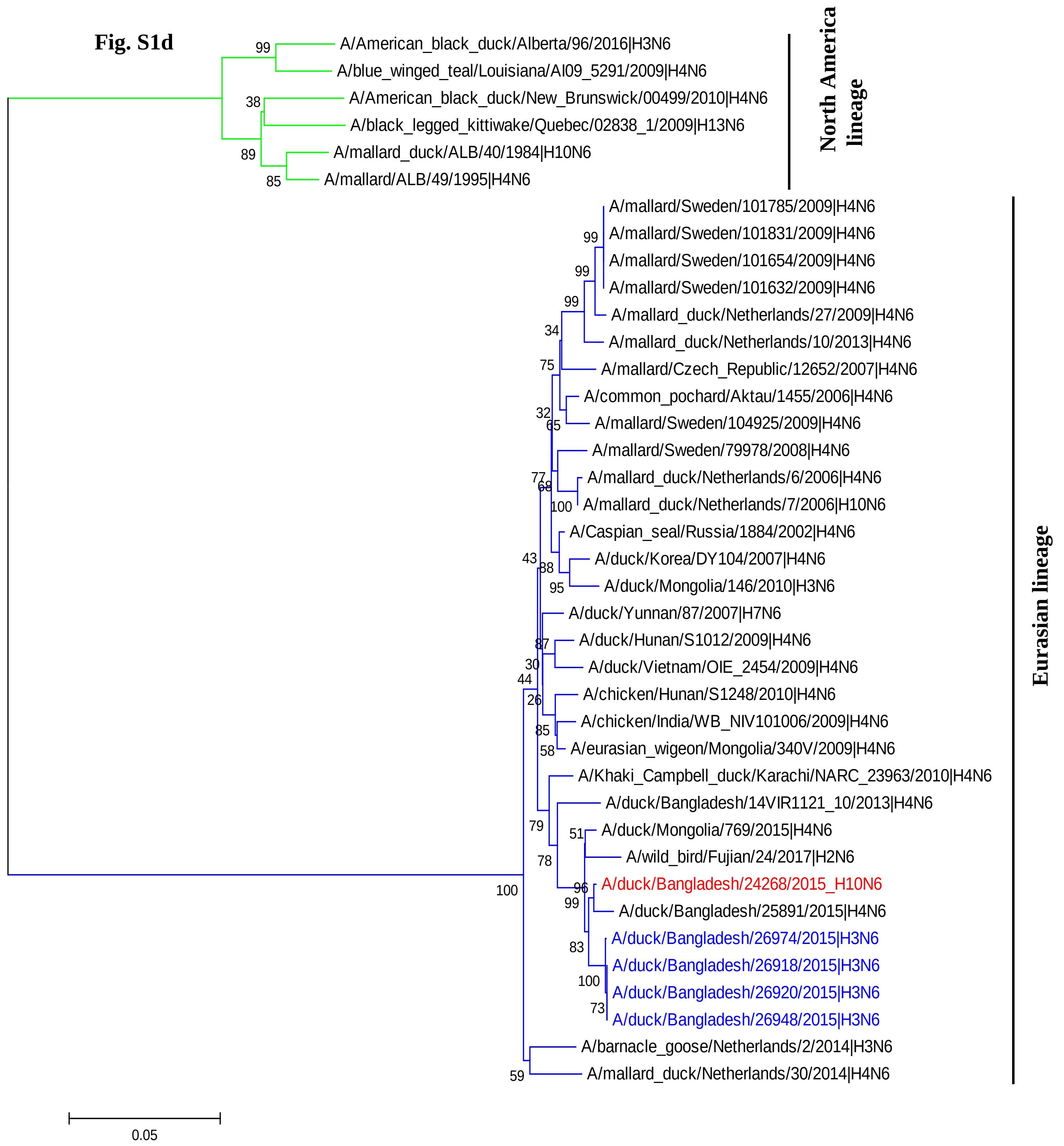
Fig. S1d
A/American_black_duck/Alberta/96/2016|H3N6
A/blue_winged_teal/Louisiana/AI09_5291/2009|H4N6
A/American_black_duck/New_Brunswick/00499/2010|H4N6
A/black_legged_kittiwake/Quebec/02838_1/2009|H13N6
A/mallard_duck/ALB/40/1984|H10N6
A/mallard/ALB/49/1995|H4N6
North America lineage
A/mallard/Sweden/101785/2009|H4N6
A/mallard/Sweden/101831/2009|H4N6
A/mallard/Sweden/101654/2009|H4N6
A/mallard/Sweden/101632/2009|H4N6
A/mallard_duck/Netherlands/27/2009|H4N6
A/mallard_duck/Netherlands/10/2013|H4N6
A/mallard/Czech_Republic/12652/2007|H4N6
A/common_pochard/Aktau/1455/2006|H4N6
A/mallard/Sweden/104925/2009|H4N6
A/mallard/Sweden/79978/2008|H4N6
A/mallard_duck/Netherlands/6/2006|H4N6
A/mallard_duck/Netherlands/7/2006|H10N6
A/Caspian_seal/Russia/1884/2002|H4N6
A/duck/Korea/DY104/2007|H4N6
A/duck/Mongolia/146/2010|H3N6
A/duck/Yunnan/87/2007|H7N6
A/duck/Hunan/S1012/2009|H4N6
A/duck/Vietnam/OIE_2454/2009|H4N6
A/chicken/Hunan/S1248/2010|H4N6
A/chicken/India/WB_NIV101006/2009|H4N6
A/eurasian_wigeon/Mongolia/340V/2009|H4N6
A/Khaki_Campbell_duck/Karachi/NARC_23963/2010|H4N6
A/duck/Bangladesh/14VIR1121_10/2013|H4N6
A/duck/Mongolia/769/2015|H4N6
A/wild_bird/Fujian/24/2017|H2N6
A/duck/Bangladesh/24268/2015_H10N6
A/duck/Bangladesh/25891/2015|H4N6
A/duck/Bangladesh/26974/2015|H3N6
A/duck/Bangladesh/26918/2015|H3N6
A/duck/Bangladesh/26920/2015|H3N6
A/duck/Bangladesh/26948/2015|H3N6
A/barnacle_goose/Netherlands/2/2014|H3N6
A/mallard_duck/Netherlands/30/2014|H4N6
Eurasian lineage
0.05

Fig. S2a

PB2

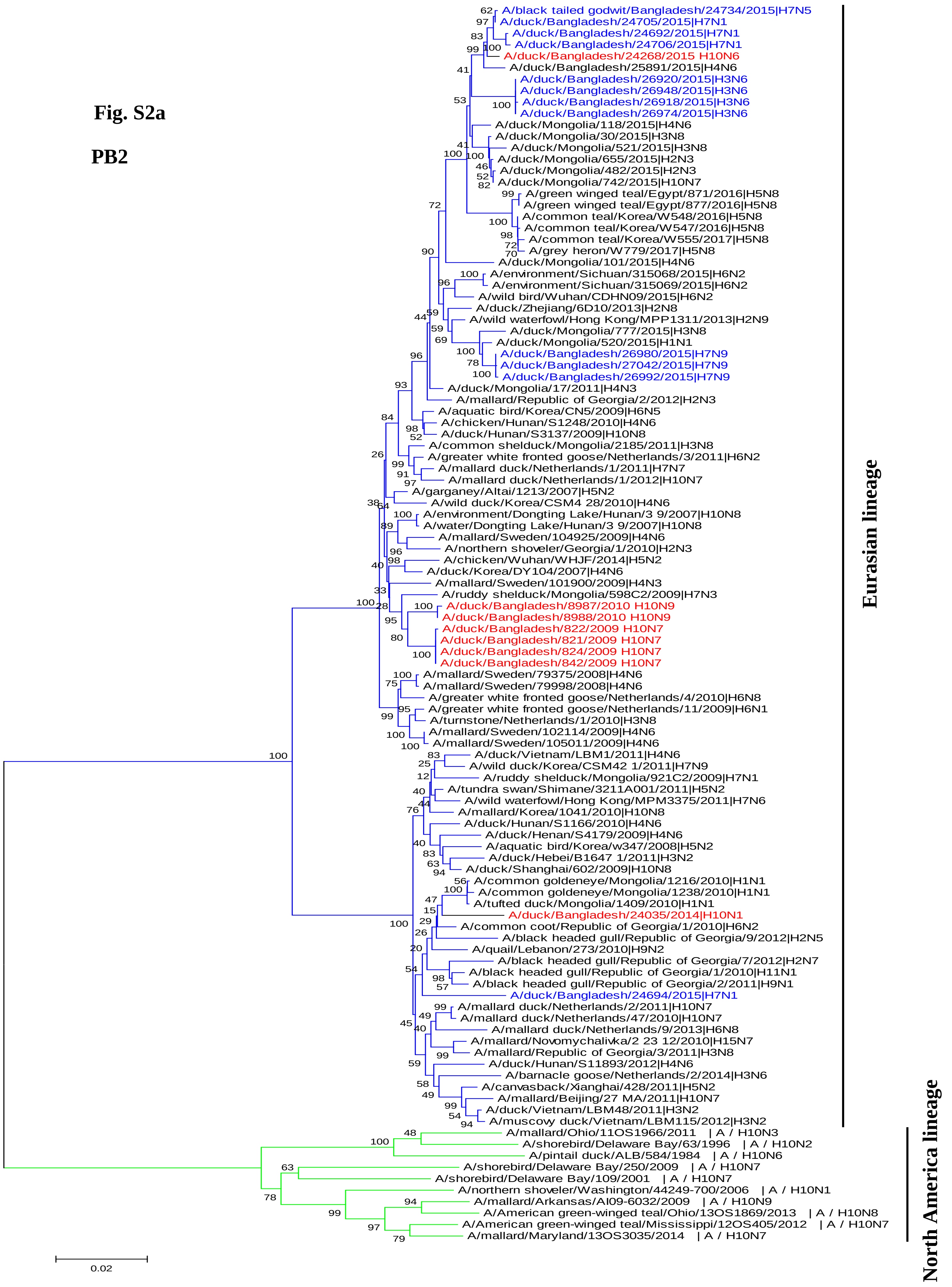
A/black tailed godwit/Bangladesh/24734/2015|H7N5
A/duck/Bangladesh/24705/2015|H7N1
A/duck/Bangladesh/24692/2015|H7N1
A/duck/Bangladesh/24706/2015|H7N1
A/duck/Bangladesh/24268/2015 H10N6
A/duck/Bangladesh/25891/2015|H4N6
A/duck/Bangladesh/26920/2015|H3N6
A/duck/Bangladesh/26948/2015|H3N6
A/duck/Bangladesh/26918/2015|H3N6
A/duck/Bangladesh/26974/2015|H3N6
A/duck/Mongolia/118/2015|H4N6
A/duck/Mongolia/30/2015|H3N8
A/duck/Mongolia/521/2015|H3N8
A/duck/Mongolia/655/2015|H2N3
A/duck/Mongolia/482/2015|H2N3
A/duck/Mongolia/742/2015|H10N7
A/green winged teal/Egypt/871/2016|H5N8
A/green winged teal/Egypt/877/2016|H5N8
A/common teal/Korea/W548/2016|H5N8
A/common teal/Korea/W547/2016|H5N8
A/common teal/Korea/W555/2017|H5N8
A/grey heron/W779/2017|H5N8
A/duck/Mongolia/101/2015|H4N6
A/environment/Sichuan/315068/2015|H6N2
A/environment/Sichuan/315069/2015|H6N2
A/wild bird/Wuhan/CDHN09/2015|H6N2
A/duck/Zhejiang/6D10/2013|H2N8
A/wild waterfowl/Hong Kong/MPP1311/2013|H2N9
A/duck/Mongolia/777/2015|H3N8
A/duck/Mongolia/520/2015|H1N1
A/duck/Bangladesh/26980/2015|H7N9
A/duck/Bangladesh/27042/2015|H7N9
A/duck/Bangladesh/26992/2015|H7N9
A/duck/Mongolia/17/2011|H4N3
A/mallard/Republic of Georgia/2/2012|H2N3
A/aquatic bird/Korea/CN5/2009|H6N5
A/chicken/Hunan/S1248/2010|H4N6
A/duck/Hunan/S3137/2009|H10N8
A/common shelduck/Mongolia/2185/2011|H3N8
A/greater white fronted goose/Netherlands/3/2011|H6N2
A/mallard duck/Netherlands/1/2011|H7N7
A/mallard duck/Netherlands/1/2012|H10N7
A/garganey/Altai/1213/2007|H5N2
A/wild duck/Korea/CSM4 28/2010|H4N6
A/environment/Dongting Lake/Hunan/3 9/2007|H10N8
A/water/Dongting Lake/Hunan/3 9/2007|H10N8
A/mallard/Sweden/104925/2009|H4N6
A/northern shoveler/Georgia/1/2010|H2N3
A/chicken/Wuhan/WHJF/2014|H5N2
A/duck/Korea/DY104/2007|H4N6
A/mallard/Sweden/101900/2009|H4N3
A/ruddy shelduck/Mongolia/598C2/2009|H7N3
A/duck/Bangladesh/8987/2010 H10N9
A/duck/Bangladesh/8988/2010 H10N9
A/duck/Bangladesh/822/2009 H10N7
A/duck/Bangladesh/821/2009 H10N7
A/duck/Bangladesh/824/2009 H10N7
A/duck/Bangladesh/842/2009 H10N7
A/mallard/Sweden/79375/2008|H4N6
A/mallard/Sweden/79998/2008|H4N6
A/greater white fronted goose/Netherlands/4/2010|H6N8
A/greater white fronted goose/Netherlands/11/2009|H6N1
A/turnstone/Netherlands/1/2010|H3N8
A/mallard/Sweden/102114/2009|H4N6
A/mallard/Sweden/105011/2009|H4N6
A/duck/Vietnam/LBM1/2011|H4N6
A/wild duck/Korea/CSM42 1/2011|H7N9
A/ruddy shelduck/Mongolia/921C2/2009|H7N1
A/tundra swan/Shimane/3211A001/2011|H5N2
A/wild waterfowl/Hong Kong/MPM3375/2011|H7N6
A/mallard/Korea/1041/2010|H10N8
A/duck/Hunan/S1166/2010|H4N6
A/duck/Henan/S4179/2009|H4N6
A/aquatic bird/Korea/w347/2008|H5N2
A/duck/Hebei/B1647 1/2011|H3N2
A/duck/Shanghai/602/2009|H10N8
A/common goldeneye/Mongolia/1216/2010|H1N1
A/common goldeneye/Mongolia/1238/2010|H1N1
A/tufted duck/Mongolia/1409/2010|H1N1
A/duck/Bangladesh/24035/2014|H10N1
A/common coot/Republic of Georgia/1/2010|H6N2
A/black headed gull/Republic of Georgia/9/2012|H2N5
A/quail/Lebanon/273/2010|H9N2
A/black headed gull/Republic of Georgia/7/2012|H2N7
A/black headed gull/Republic of Georgia/1/2010|H11N1
A/black headed gull/Republic of Georgia/2/2011|H9N1
A/duck/Bangladesh/24694/2015|H7N1
A/mallard duck/Netherlands/2/2011|H10N7
A/mallard duck/Netherlands/47/2010|H10N7
A/mallard duck/Netherlands/9/2013|H6N8
A/mallard/Novomychalivka/2 23 12/2010|H15N7
A/mallard/Republic of Georgia/3/2011|H3N8
A/duck/Hunan/S11893/2012|H4N6
A/barnacle goose/Netherlands/2/2014|H3N6
A/canvasback/Xianghai/428/2011|H5N2
A/mallard/Beijing/27 MA/2011|H10N7
A/duck/Vietnam/LBM48/2011|H3N2
A/muscovy duck/Vietnam/LBM115/2012|H3N2
A/mallard/Ohio/11OS1966/2011 | A / H10N3
A/shorebird/Delaware Bay/63/1996 | A / H10N2
A/pintail duck/ALB/584/1984 | A / H10N6
A/shorebird/Delaware Bay/250/2009 | A / H10N7
A/shorebird/Delaware Bay/109/2001 | A / H10N7
A/northern shoveler/Washington/44249-700/2006 | A / H10N1
A/mallard/Arkansas/AI09-6032/2009 | A / H10N9
A/American green-winged teal/Ohio/13OS1869/2013 | A / H10N8
A/American green-winged teal/Mississippi/12OS405/2012 | A / H10N7
A/mallard/Maryland/13OS3035/2014 | A / H10N7
Eurasian lineage
North America lineage
0.02

Fig. S2b

PB1

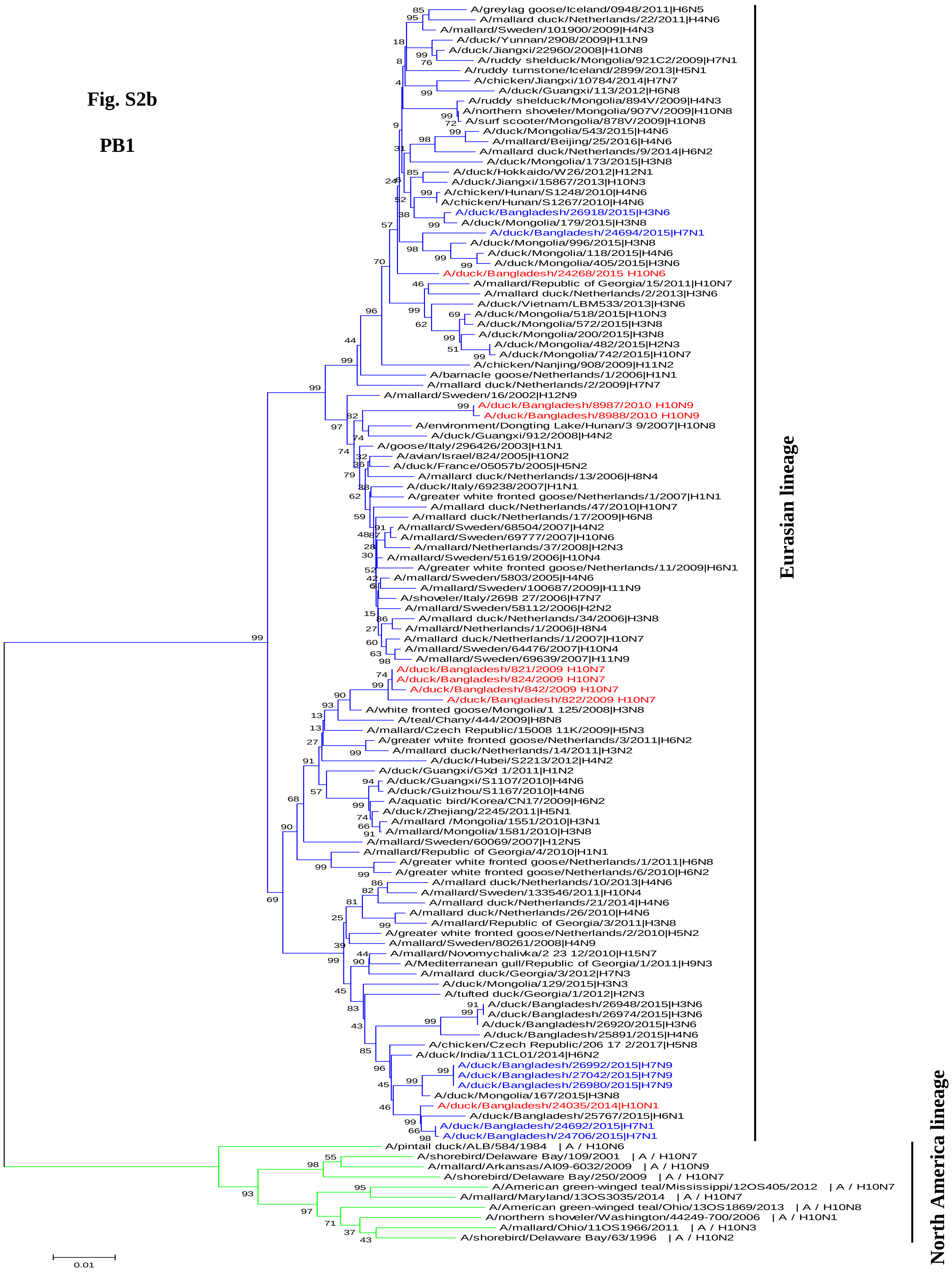
A/greylag goose/Iceland/0948/2011|H6N5
A/mallard duck/Netherlands/22/2011|H4N6
A/mallard/Sweden/101900/2009|H4N3
A/duck/Yunnan/2908/2009|H11N9
A/duck/Jiangxi/22960/2008|H10N8
A/ruddy shelduck/Mongolia/921C2/2009|H7N1
A/ruddy turnstone/Iceland/2899/2013|H5N1
A/chicken/Jiangxi/10784/2014|H7N7
A/duck/Guangxi/113/2012|H6N8
A/ruddy shelduck/Mongolia/894V/2009|H4N3
A/northern shoveler/Mongolia/907V/2009|H10N8
A/surf scooter/Mongolia/878V/2009|H10N8
A/duck/Mongolia/543/2015|H4N6
A/mallard/Beijing/25/2016|H4N6
A/mallard duck/Netherlands/9/2014|H6N2
A/duck/Mongolia/173/2015|H3N8
A/duck/Hokkaido/W26/2012|H12N1
A/duck/Jiangxi/15867/2013|H10N3
A/chicken/Hunan/S1248/2010|H4N6
A/chicken/Hunan/S1267/2010|H4N6
A/duck/Bangladesh/26918/2015|H3N6
A/duck/Mongolia/179/2015|H3N8
A/duck/Bangladesh/24694/2015|H7N1
A/duck/Mongolia/996/2015|H3N8
A/duck/Mongolia/118/2015|H4N6
A/duck/Mongolia/405/2015|H3N6
A/duck/Bangladesh/24268/2015 H10N6
A/mallard/Republic of Georgia/15/2011|H10N7
A/mallard duck/Netherlands/2/2013|H3N6
A/duck/Vietnam/LBM533/2013|H3N6
A/duck/Mongolia/518/2015|H10N3
A/duck/Mongolia/572/2015|H3N8
A/duck/Mongolia/200/2015|H3N8
A/duck/Mongolia/482/2015|H2N3
A/duck/Mongolia/742/2015|H10N7
A/chicken/Nanjing/908/2009|H11N2
A/barnacle goose/Netherlands/1/2006|H1N1
A/mallard duck/Netherlands/2/2009|H7N7
A/mallard/Sweden/16/2002|H12N9
A/duck/Bangladesh/8987/2010 H10N9
A/duck/Bangladesh/8988/2010 H10N9
A/environment/Dongting Lake/Hunan/3 9/2007|H10N8
A/duck/Guangxi/912/2008|H4N2
A/goose/Italy/296426/2003|H1N1
A/avian/Israel/824/2005|H10N2
A/duck/France/05057b/2005|H5N2
A/mallard duck/Netherlands/13/2006|H8N4
A/duck/Italy/69238/2007|H1N1
A/greater white fronted goose/Netherlands/1/2007|H1N1
A/mallard duck/Netherlands/47/2010|H10N7
A/mallard duck/Netherlands/17/2009|H6N8
A/mallard/Sweden/68504/2007|H4N2
A/mallard/Sweden/69777/2007|H10N6
A/mallard/Netherlands/37/2008|H2N3
A/mallard/Sweden/51619/2006|H10N4
A/greater white fronted goose/Netherlands/11/2009|H6N1
A/mallard/Sweden/5803/2005|H4N6
A/mallard/Sweden/100687/2009|H11N9
A/shoveler/Italy/2698 27/2006|H7N7
A/mallard/Sweden/58112/2006|H2N2
A/mallard duck/Netherlands/34/2006|H3N8
A/mallard/Netherlands/1/2006|H8N4
A/mallard duck/Netherlands/1/2007|H10N7
A/mallard/Sweden/64476/2007|H10N4
A/mallard/Sweden/69639/2007|H11N9
A/duck/Bangladesh/821/2009 H10N7
A/duck/Bangladesh/824/2009 H10N7
A/duck/Bangladesh/842/2009 H10N7
A/duck/Bangladesh/822/2009 H10N7
A/white fronted goose/Mongolia/1 125/2008|H3N8
A/teal/Chany/444/2009|H8N8
A/mallard/Czech Republic/15008 11K/2009|H5N3
A/greater white fronted goose/Netherlands/3/2011|H6N2
A/mallard duck/Netherlands/14/2011|H3N2
A/duck/Hubei/S2213/2012|H4N2
A/duck/Guangxi/GXd 1/2011|H1N2
A/duck/Guangxi/S1107/2010|H4N6
A/duck/Guizhou/S1167/2010|H4N6
A/aquatic bird/Korea/CN17/2009|H6N2
A/duck/Zhejiang/2245/2011|H5N1
A/mallard /Mongolia/1551/2010|H3N1
A/mallard/Mongolia/1581/2010|H3N8
A/mallard/Sweden/60069/2007|H12N5
A/mallard/Republic of Georgia/4/2010|H1N1
A/greater white fronted goose/Netherlands/1/2011|H6N8
A/greater white fronted goose/Netherlands/6/2010|H6N2
A/mallard duck/Netherlands/10/2013|H4N6
A/mallard/Sweden/133546/2011|H10N4
A/mallard duck/Netherlands/21/2014|H4N6
A/mallard duck/Netherlands/26/2010|H4N6
A/mallard/Republic of Georgia/3/2011|H3N8
A/greater white fronted goose/Netherlands/2/2010|H5N2
A/mallard/Sweden/80261/2008|H4N9
A/mallard/Novomychalivka/2 23 12/2010|H15N7
A/Mediterranean gull/Republic of Georgia/1/2011|H9N3
A/mallard duck/Georgia/3/2012|H7N3
A/duck/Mongolia/129/2015|H3N3
A/tufted duck/Georgia/1/2012|H2N3
A/duck/Bangladesh/26948/2015|H3N6
A/duck/Bangladesh/26974/2015|H3N6
A/duck/Bangladesh/26920/2015|H3N6
A/duck/Bangladesh/25891/2015|H4N6
A/chicken/Czech Republic/206 17 2/2017|H5N8
A/duck/India/11CL01/2014|H6N2
A/duck/Bangladesh/26992/2015|H7N9
A/duck/Bangladesh/27042/2015|H7N9
A/duck/Bangladesh/26980/2015|H7N9
A/duck/Mongolia/167/2015|H3N8
A/duck/Bangladesh/24035/2014|H10N1
A/duck/Bangladesh/25767/2015|H6N1
A/duck/Bangladesh/24692/2015|H7N1
A/duck/Bangladesh/24706/2015|H7N1
A/pintail duck/ALB/584/1984 | A / H10N6
A/shorebird/Delaware Bay/109/2001 | A / H10N7
A/mallard/Arkansas/AI09-6032/2009 | A / H10N9
A/shorebird/Delaware Bay/250/2009 | A / H10N7
A/American green-winged teal/Mississippi/12OS405/2012 | A / H10N7
A/mallard/Maryland/13OS3035/2014 | A / H10N7
A/American green-winged teal/Ohio/13OS1869/2013 | A / H10N8
A/northern shoveler/Washington/44249-700/2006 | A / H10N1
A/mallard/Ohio/11OS1966/2011 | A / H10N3
A/shorebird/Delaware Bay/63/1996 | A / H10N2
Eurasian lineage
North America lineage
0.01

Fig. S2c

PA

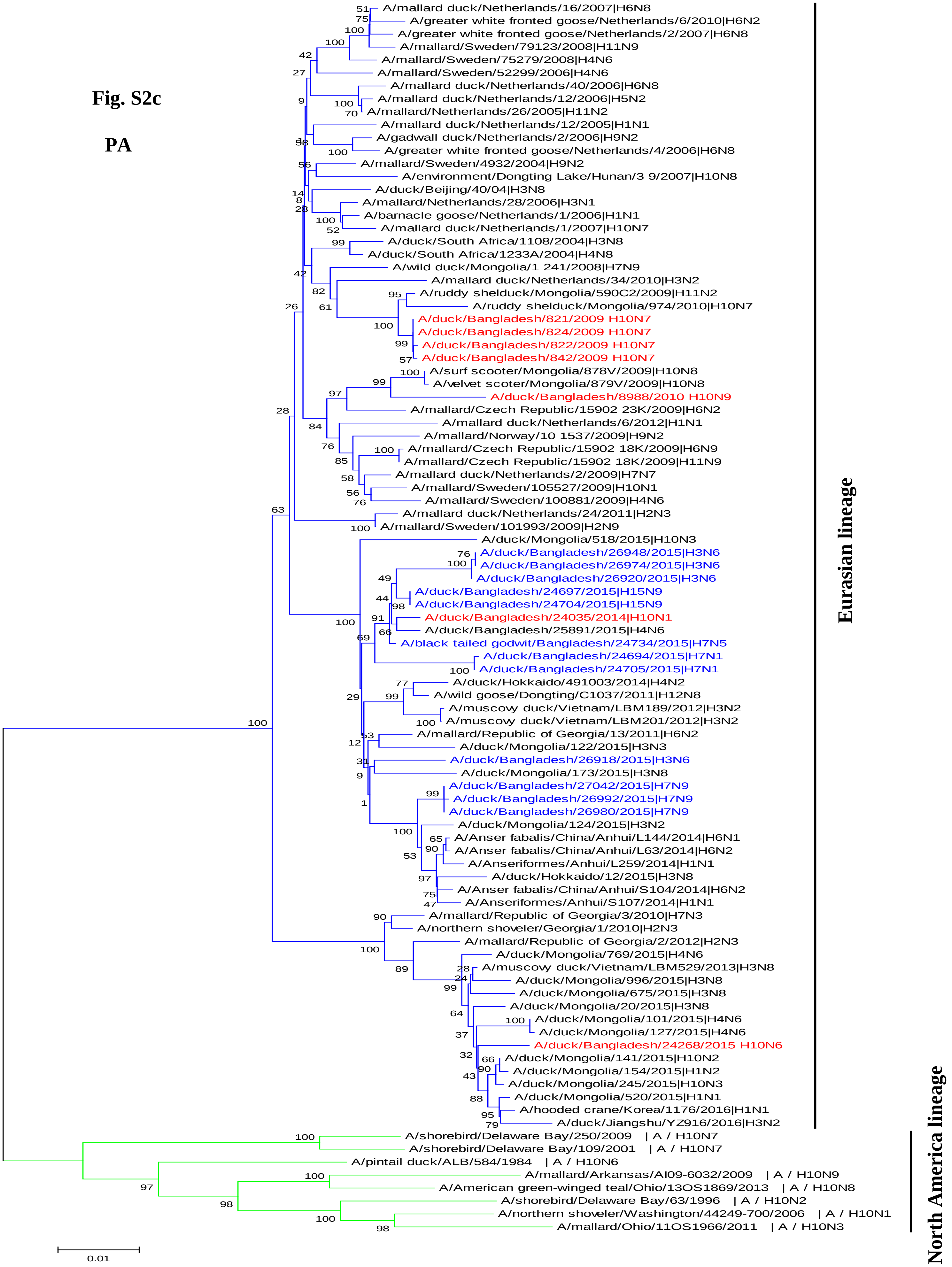
A/mallard duck/Netherlands/16/2007|H6N8
A/greater white fronted goose/Netherlands/6/2010|H6N2
A/greater white fronted goose/Netherlands/2/2007|H6N8
A/mallard/Sweden/79123/2008|H11N9
A/mallard/Sweden/75279/2008|H4N6
A/mallard/Sweden/52299/2006|H4N6
A/mallard duck/Netherlands/40/2006|H6N8
A/mallard duck/Netherlands/12/2006|H5N2
A/mallard/Netherlands/26/2005|H11N2
A/mallard duck/Netherlands/12/2005|H1N1
A/gadwall duck/Netherlands/2/2006|H9N2
A/greater white fronted goose/Netherlands/4/2006|H6N8
A/mallard/Sweden/4932/2004|H9N2
A/environment/Dongting Lake/Hunan/3 9/2007|H10N8
A/duck/Beijing/40/04|H3N8
A/mallard/Netherlands/28/2006|H3N1
A/barnacle goose/Netherlands/1/2006|H1N1
A/mallard duck/Netherlands/1/2007|H10N7
A/duck/South Africa/1108/2004|H3N8
A/duck/South Africa/1233A/2004|H4N8
A/wild duck/Mongolia/1 241/2008|H7N9
A/mallard duck/Netherlands/34/2010|H3N2
A/ruddy shelduck/Mongolia/590C2/2009|H11N2
A/ruddy shelduck/Mongolia/974/2010|H10N7
A/duck/Bangladesh/821/2009 H10N7
A/duck/Bangladesh/824/2009 H10N7
A/duck/Bangladesh/822/2009 H10N7
A/duck/Bangladesh/842/2009 H10N7
A/surf scooter/Mongolia/878V/2009|H10N8
A/velvet scoter/Mongolia/879V/2009|H10N8
A/duck/Bangladesh/8988/2010 H10N9
A/mallard/Czech Republic/15902 23K/2009|H6N2
A/mallard duck/Netherlands/6/2012|H1N1
A/mallard/Norway/10 1537/2009|H9N2
A/mallard/Czech Republic/15902 18K/2009|H6N9
A/mallard/Czech Republic/15902 18K/2009|H11N9
A/mallard duck/Netherlands/2/2009|H7N7
A/mallard/Sweden/105527/2009|H10N1
A/mallard/Sweden/100881/2009|H4N6
A/mallard duck/Netherlands/24/2011|H2N3
A/mallard/Sweden/101993/2009|H2N9
A/duck/Mongolia/518/2015|H10N3
A/duck/Bangladesh/26948/2015|H3N6
A/duck/Bangladesh/26974/2015|H3N6
A/duck/Bangladesh/26920/2015|H3N6
A/duck/Bangladesh/24697/2015|H15N9
A/duck/Bangladesh/24704/2015|H15N9
A/duck/Bangladesh/24035/2014|H10N1
A/duck/Bangladesh/25891/2015|H4N6
A/black tailed godwit/Bangladesh/24734/2015|H7N5
A/duck/Bangladesh/24694/2015|H7N1
A/duck/Bangladesh/24705/2015|H7N1
A/duck/Hokkaido/491003/2014|H4N2
A/wild goose/Dongting/C1037/2011|H12N8
A/muscovy duck/Vietnam/LBM189/2012|H3N2
A/muscovy duck/Vietnam/LBM201/2012|H3N2
A/mallard/Republic of Georgia/13/2011|H6N2
A/duck/Mongolia/122/2015|H3N3
A/duck/Bangladesh/26918/2015|H3N6
A/duck/Mongolia/173/2015|H3N8
A/duck/Bangladesh/27042/2015|H7N9
A/duck/Bangladesh/26992/2015|H7N9
A/duck/Bangladesh/26980/2015|H7N9
A/duck/Mongolia/124/2015|H3N2
A/Anser fabalis/China/Anhui/L144/2014|H6N1
A/Anser fabalis/China/Anhui/L63/2014|H6N2
A/Anseriformes/Anhui/L259/2014|H1N1
A/duck/Hokkaido/12/2015|H3N8
A/Anser fabalis/China/Anhui/S104/2014|H6N2
A/Anseriformes/Anhui/S107/2014|H1N1
A/mallard/Republic of Georgia/3/2010|H7N3
A/northern shoveler/Georgia/1/2010|H2N3
A/mallard/Republic of Georgia/2/2012|H2N3
A/duck/Mongolia/769/2015|H4N6
A/muscovy duck/Vietnam/LBM529/2013|H3N8
A/duck/Mongolia/996/2015|H3N8
A/duck/Mongolia/675/2015|H3N8
A/duck/Mongolia/20/2015|H3N8
A/duck/Mongolia/101/2015|H4N6
A/duck/Mongolia/127/2015|H4N6
A/duck/Bangladesh/24268/2015 H10N6
A/duck/Mongolia/141/2015|H10N2
A/duck/Mongolia/154/2015|H1N2
A/duck/Mongolia/245/2015|H10N3
A/duck/Mongolia/520/2015|H1N1
A/hooded crane/Korea/1176/2016|H1N1
A/duck/Jiangshu/YZ916/2016|H3N2
A/shorebird/Delaware Bay/250/2009 | A / H10N7
A/shorebird/Delaware Bay/109/2001 | A / H10N7
A/pintail duck/ALB/584/1984 | A / H10N6
A/mallard/Arkansas/AI09-6032/2009 | A / H10N9
A/American green-winged teal/Ohio/13OS1869/2013 | A / H10N8
A/shorebird/Delaware Bay/63/1996 | A / H10N2
A/northern shoveler/Washington/44249-700/2006 | A / H10N1
A/mallard/Ohio/11OS1966/2011 | A / H10N3
Eurasian lineage
North America lineage
0.01

Fig. S2d

NP

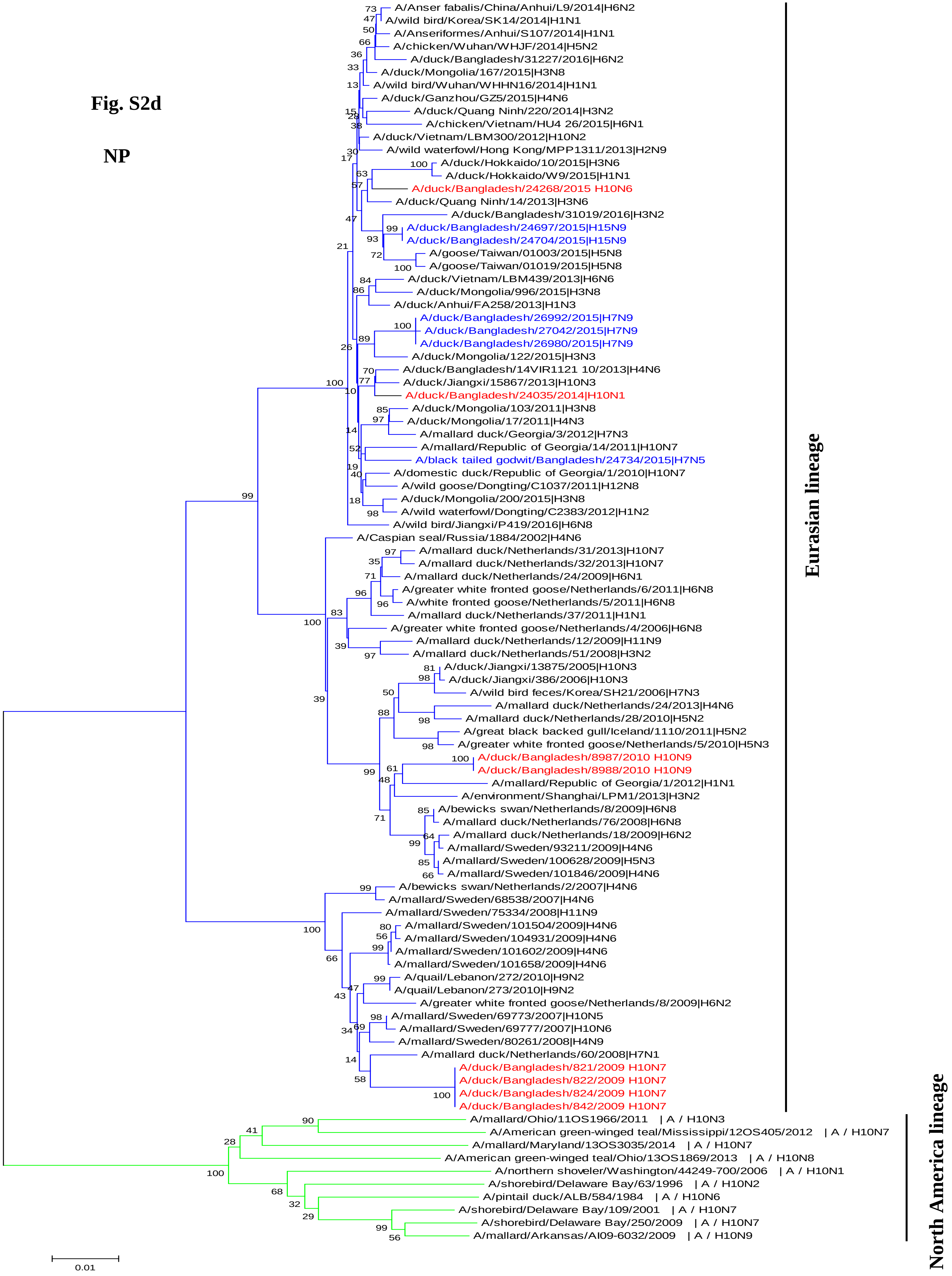
A/Anser fabalis/China/Anhui/L9/2014|H6N2
A/wild bird/Korea/SK14/2014|H1N1
A/Anseriformes/Anhui/S107/2014|H1N1
A/chicken/Wuhan/WHJF/2014|H5N2
A/duck/Bangladesh/31227/2016|H6N2
A/duck/Mongolia/167/2015|H3N8
A/wild bird/Wuhan/WHHN16/2014|H1N1
A/duck/Ganzhou/GZ5/2015|H4N6
A/duck/Quang Ninh/220/2014|H3N2
A/chicken/Vietnam/HU4 26/2015|H6N1
A/duck/Vietnam/LBM300/2012|H10N2
A/wild waterfowl/Hong Kong/MPP1311/2013|H2N9
A/duck/Hokkaido/10/2015|H3N6
A/duck/Hokkaido/W9/2015|H1N1
A/duck/Bangladesh/24268/2015 H10N6
A/duck/Quang Ninh/14/2013|H3N6
A/duck/Bangladesh/31019/2016|H3N2
A/duck/Bangladesh/24697/2015|H15N9
A/duck/Bangladesh/24704/2015|H15N9
A/goose/Taiwan/01003/2015|H5N8
A/goose/Taiwan/01019/2015|H5N8
A/duck/Vietnam/LBM439/2013|H6N6
A/duck/Mongolia/996/2015|H3N8
A/duck/Anhui/FA258/2013|H1N3
A/duck/Bangladesh/26992/2015|H7N9
A/duck/Bangladesh/27042/2015|H7N9
A/duck/Bangladesh/26980/2015|H7N9
A/duck/Mongolia/122/2015|H3N3
A/duck/Bangladesh/14VIR1121 10/2013|H4N6
A/duck/Jiangxi/15867/2013|H10N3
A/duck/Bangladesh/24035/2014|H10N1
A/duck/Mongolia/103/2011|H3N8
A/duck/Mongolia/17/2011|H4N3
A/mallard duck/Georgia/3/2012|H7N3
A/mallard/Republic of Georgia/14/2011|H10N7
A/black tailed godwit/Bangladesh/24734/2015|H7N5
A/domestic duck/Republic of Georgia/1/2010|H10N7
A/wild goose/Dongting/C1037/2011|H12N8
A/duck/Mongolia/200/2015|H3N8
A/wild waterfowl/Dongting/C2383/2012|H1N2
A/wild bird/Jiangxi/P419/2016|H6N8
A/Caspian seal/Russia/1884/2002|H4N6
A/mallard duck/Netherlands/31/2013|H10N7
A/mallard duck/Netherlands/32/2013|H10N7
A/mallard duck/Netherlands/24/2009|H6N1
A/greater white fronted goose/Netherlands/6/2011|H6N8
A/white fronted goose/Netherlands/5/2011|H6N8
A/mallard duck/Netherlands/37/2011|H1N1
A/greater white fronted goose/Netherlands/4/2006|H6N8
A/mallard duck/Netherlands/12/2009|H11N9
A/mallard duck/Netherlands/51/2008|H3N2
A/duck/Jiangxi/13875/2005|H10N3
A/duck/Jiangxi/386/2006|H10N3
A/wild bird feces/Korea/SH21/2006|H7N3
A/mallard duck/Netherlands/24/2013|H4N6
A/mallard duck/Netherlands/28/2010|H5N2
A/great black backed gull/Iceland/1110/2011|H5N2
A/greater white fronted goose/Netherlands/5/2010|H5N3
A/duck/Bangladesh/8987/2010 H10N9
A/duck/Bangladesh/8988/2010 H10N9
A/mallard/Republic of Georgia/1/2012|H1N1
A/environment/Shanghai/LPM1/2013|H3N2
A/bewicks swan/Netherlands/8/2009|H6N8
A/mallard duck/Netherlands/76/2008|H6N8
A/mallard duck/Netherlands/18/2009|H6N2
A/mallard/Sweden/93211/2009|H4N6
A/mallard/Sweden/100628/2009|H5N3
A/mallard/Sweden/101846/2009|H4N6
A/bewicks swan/Netherlands/2/2007|H4N6
A/mallard/Sweden/68538/2007|H4N6
A/mallard/Sweden/75334/2008|H11N9
A/mallard/Sweden/101504/2009|H4N6
A/mallard/Sweden/104931/2009|H4N6
A/mallard/Sweden/101602/2009|H4N6
A/mallard/Sweden/101658/2009|H4N6
A/quail/Lebanon/272/2010|H9N2
A/quail/Lebanon/273/2010|H9N2
A/greater white fronted goose/Netherlands/8/2009|H6N2
A/mallard/Sweden/69773/2007|H10N5
A/mallard/Sweden/69777/2007|H10N6
A/mallard/Sweden/80261/2008|H4N9
A/mallard duck/Netherlands/60/2008|H7N1
A/duck/Bangladesh/821/2009 H10N7
A/duck/Bangladesh/822/2009 H10N7
A/duck/Bangladesh/824/2009 H10N7
A/duck/Bangladesh/842/2009 H10N7
A/mallard/Ohio/11OS1966/2011 | A / H10N3
A/American green-winged teal/Mississippi/12OS405/2012 | A / H10N7
A/mallard/Maryland/13OS3035/2014 | A / H10N7
A/American green-winged teal/Ohio/13OS1869/2013 | A / H10N8
A/northern shoveler/Washington/44249-700/2006 | A / H10N1
A/shorebird/Delaware Bay/63/1996 | A / H10N2
A/pintail duck/ALB/584/1984 | A / H10N6
A/shorebird/Delaware Bay/109/2001 | A / H10N7
A/shorebird/Delaware Bay/250/2009 | A / H10N7
A/mallard/Arkansas/AI09-6032/2009 | A / H10N9
Eurasian lineage
North America lineage
0.01

Fig. S2e

M

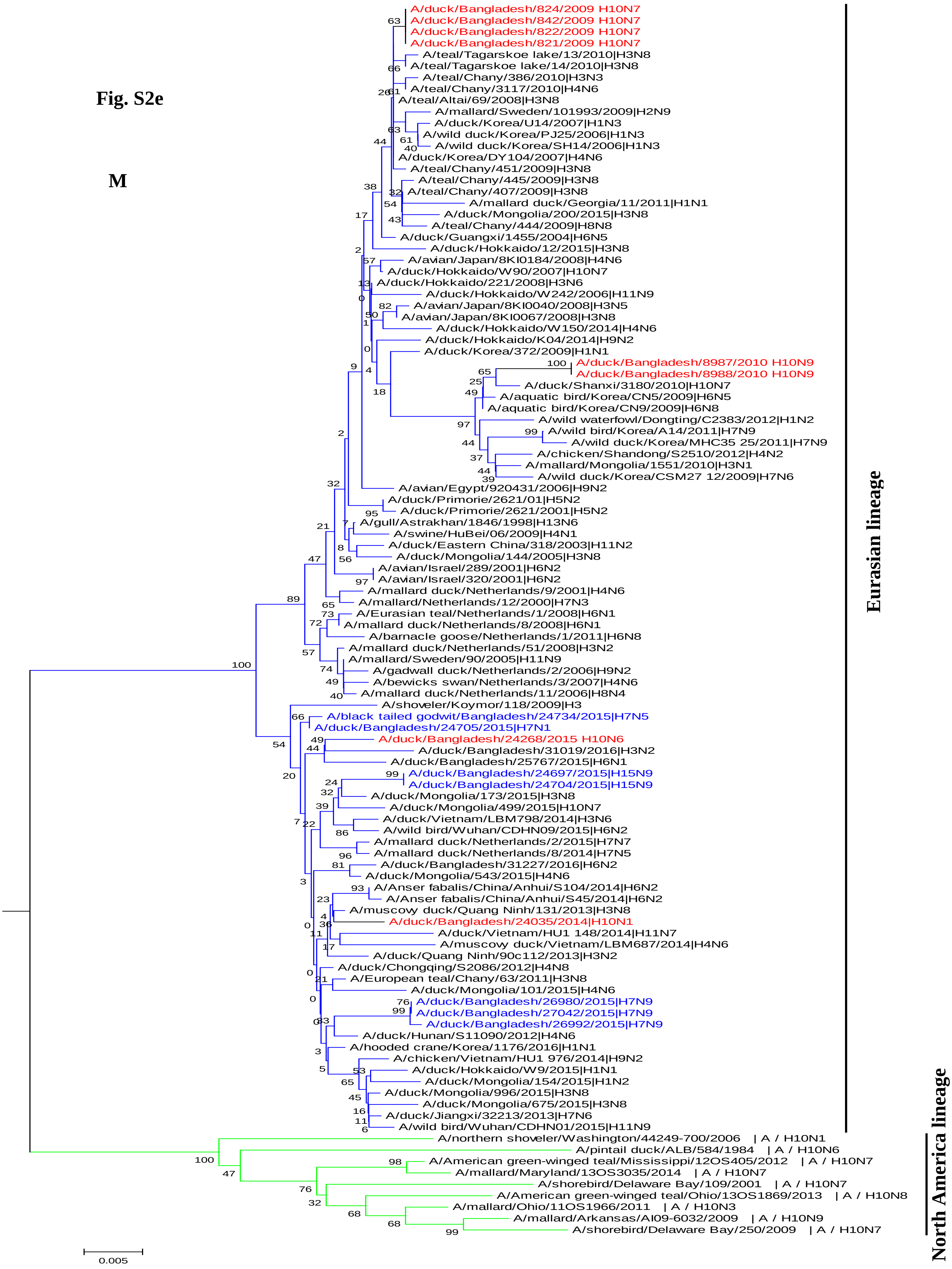

A/duck/Bangladesh/824/2009 H10N7
A/duck/Bangladesh/842/2009 H10N7
A/duck/Bangladesh/822/2009 H10N7
A/duck/Bangladesh/821/2009 H10N7
A/teal/Tagarskoe lake/13/2010|H3N8
A/teal/Tagarskoe lake/14/2010|H3N8
A/teal/Chany/386/2010|H3N3
A/teal/Chany/3117/2010|H4N6
A/teal/Altai/69/2008|H3N8
A/mallard/Sweden/101993/2009|H2N9
A/duck/Korea/U14/2007|H1N3
A/wild duck/Korea/PJ25/2006|H1N3
A/wild duck/Korea/SH14/2006|H1N3
A/duck/Korea/DY104/2007|H4N6
A/teal/Chany/451/2009|H3N8
A/teal/Chany/445/2009|H3N8
A/teal/Chany/407/2009|H3N8
A/mallard duck/Georgia/11/2011|H1N1
A/duck/Mongolia/200/2015|H3N8
A/teal/Chany/444/2009|H8N8
A/duck/Guangxi/1455/2004|H6N5
A/duck/Hokkaido/12/2015|H3N8
A/avian/Japan/8KI0184/2008|H4N6
A/duck/Hokkaido/W90/2007|H10N7
A/duck/Hokkaido/221/2008|H3N6
A/duck/Hokkaido/W242/2006|H11N9
A/avian/Japan/8KI0040/2008|H3N5
A/avian/Japan/8KI0067/2008|H3N8
A/duck/Hokkaido/W150/2014|H4N6
A/duck/Hokkaido/K04/2014|H9N2
A/duck/Korea/372/2009|H1N1
A/duck/Bangladesh/8987/2010 H10N9
A/duck/Bangladesh/8988/2010 H10N9
A/duck/Shanxi/3180/2010|H10N7
A/aquatic bird/Korea/CN5/2009|H6N5
A/aquatic bird/Korea/CN9/2009|H6N8
A/wild waterfowl/Dongting/C2383/2012|H1N2
A/wild bird/Korea/A14/2011|H7N9
A/wild duck/Korea/MHC35 25/2011|H7N9
A/chicken/Shandong/S2510/2012|H4N2
A/mallard/Mongolia/1551/2010|H3N1
A/wild duck/Korea/CSM27 12/2009|H7N6
A/avian/Egypt/920431/2006|H9N2
A/duck/Primorie/2621/01|H5N2
A/duck/Primorie/2621/2001|H5N2
A/gull/Astrakhan/1846/1998|H13N6
A/swine/HuBei/06/2009|H4N1
A/duck/Eastern China/318/2003|H11N2
A/duck/Mongolia/144/2005|H3N8
A/avian/Israel/289/2001|H6N2
A/avian/Israel/320/2001|H6N2
A/mallard duck/Netherlands/9/2001|H4N6
A/mallard/Netherlands/12/2000|H7N3
A/Eurasian teal/Netherlands/1/2008|H6N1
A/mallard duck/Netherlands/8/2008|H6N1
A/barnacle goose/Netherlands/1/2011|H6N8
A/mallard duck/Netherlands/51/2008|H3N2
A/mallard/Sweden/90/2005|H11N9
A/gadwall duck/Netherlands/2/2006|H9N2
A/bewicks swan/Netherlands/3/2007|H4N6
A/mallard duck/Netherlands/11/2006|H8N4
A/shoveler/Koymor/118/2009|H3
A/black tailed godwit/Bangladesh/24734/2015|H7N5
A/duck/Bangladesh/24705/2015|H7N1
A/duck/Bangladesh/24268/2015 H10N6
A/duck/Bangladesh/31019/2016|H3N2
A/duck/Bangladesh/25767/2015|H6N1
A/duck/Bangladesh/24697/2015|H15N9
A/duck/Bangladesh/24704/2015|H15N9
A/duck/Mongolia/173/2015|H3N8
A/duck/Mongolia/499/2015|H10N7
A/duck/Vietnam/LBM798/2014|H3N6
A/wild bird/Wuhan/CDHN09/2015|H6N2
A/mallard duck/Netherlands/2/2015|H7N7
A/mallard duck/Netherlands/8/2014|H7N5
A/duck/Bangladesh/31227/2016|H6N2
A/duck/Mongolia/543/2015|H4N6
A/Anser fabalis/China/Anhui/S104/2014|H6N2
A/Anser fabalis/China/Anhui/S45/2014|H6N2
A/muscovy duck/Quang Ninh/131/2013|H3N8
A/duck/Bangladesh/24035/2014|H10N1
A/duck/Vietnam/HU1 148/2014|H11N7
A/muscovy duck/Vietnam/LBM687/2014|H4N6
A/duck/Quang Ninh/90c112/2013|H3N2
A/duck/Chongqing/S2086/2012|H4N8
A/European teal/Chany/63/2011|H3N8
A/duck/Mongolia/101/2015|H4N6
A/duck/Bangladesh/26980/2015|H7N9
A/duck/Bangladesh/27042/2015|H7N9
A/duck/Bangladesh/26992/2015|H7N9
A/duck/Hunan/S11090/2012|H4N6
A/hooded crane/Korea/1176/2016|H1N1
A/chicken/Vietnam/HU1 976/2014|H9N2
A/duck/Hokkaido/W9/2015|H1N1
A/duck/Mongolia/154/2015|H1N2
A/duck/Mongolia/996/2015|H3N8
A/duck/Mongolia/675/2015|H3N8
A/duck/Jiangxi/32213/2013|H7N6
A/wild bird/Wuhan/CDHN01/2015|H11N9
A/northern shoveler/Washington/44249-700/2006 | A / H10N1
A/pintail duck/ALB/584/1984 | A / H10N6
A/American green-winged teal/Mississippi/12OS405/2012 | A / H10N7
A/mallard/Maryland/13OS3035/2014 | A / H10N7
A/shorebird/Delaware Bay/109/2001 | A / H10N7
A/American green-winged teal/Ohio/13OS1869/2013 | A / H10N8
A/mallard/Ohio/11OS1966/2011 | A / H10N3
A/mallard/Arkansas/AI09-6032/2009 | A / H10N9
A/shorebird/Delaware Bay/250/2009 | A / H10N7
Eurasian lineage
North America lineage
0.005

Fig. S2f

NS

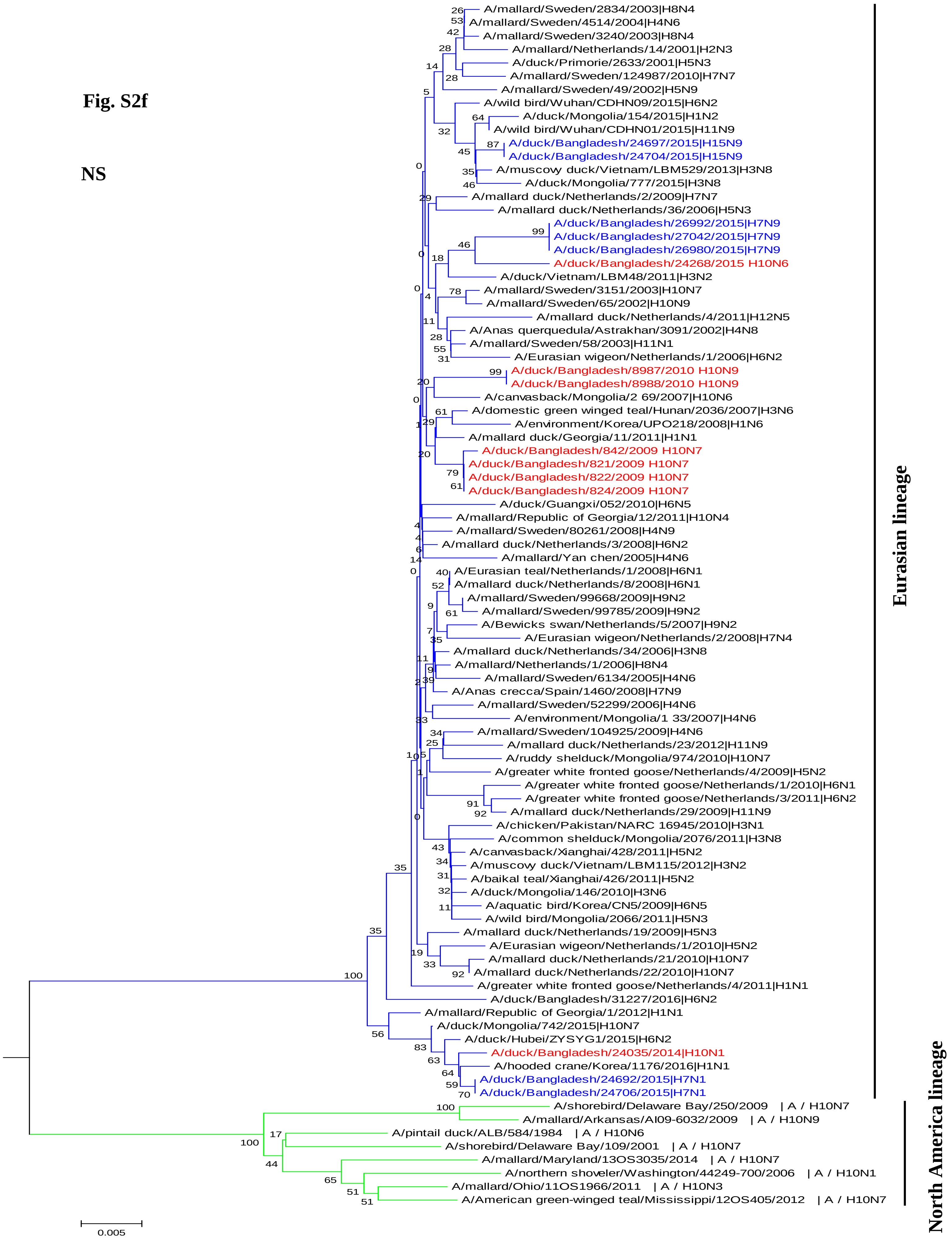

A/mallard/Sweden/2834/2003|H8N4
A/mallard/Sweden/4514/2004|H4N6
A/mallard/Sweden/3240/2003|H8N4
A/mallard/Netherlands/14/2001|H2N3
A/duck/Primorie/2633/2001|H5N3
A/mallard/Sweden/124987/2010|H7N7
A/mallard/Sweden/49/2002|H5N9
A/wild bird/Wuhan/CDHN09/2015|H6N2
A/duck/Mongolia/154/2015|H1N2
A/wild bird/Wuhan/CDHN01/2015|H11N9
A/duck/Bangladesh/24697/2015|H15N9
A/duck/Bangladesh/24704/2015|H15N9
A/muscovy duck/Vietnam/LBM529/2013|H3N8
A/duck/Mongolia/777/2015|H3N8
A/mallard duck/Netherlands/2/2009|H7N7
A/mallard duck/Netherlands/36/2006|H5N3
A/duck/Bangladesh/26992/2015|H7N9
A/duck/Bangladesh/27042/2015|H7N9
A/duck/Bangladesh/26980/2015|H7N9
A/duck/Bangladesh/24268/2015 H10N6
A/duck/Vietnam/LBM48/2011|H3N2
A/mallard/Sweden/3151/2003|H10N7
A/mallard/Sweden/65/2002|H10N9
A/mallard duck/Netherlands/4/2011|H12N5
A/Anas querquedula/Astrakhan/3091/2002|H4N8
A/mallard/Sweden/58/2003|H11N1
A/Eurasian wigeon/Netherlands/1/2006|H6N2
A/duck/Bangladesh/8987/2010 H10N9
A/duck/Bangladesh/8988/2010 H10N9
A/canvasback/Mongolia/2 69/2007|H10N6
A/domestic green winged teal/Hunan/2036/2007|H3N6
A/environment/Korea/UPO218/2008|H1N6
A/mallard duck/Georgia/11/2011|H1N1
A/duck/Bangladesh/842/2009 H10N7
A/duck/Bangladesh/821/2009 H10N7
A/duck/Bangladesh/822/2009 H10N7
A/duck/Bangladesh/824/2009 H10N7
A/duck/Guangxi/052/2010|H6N5
A/mallard/Republic of Georgia/12/2011|H10N4
A/mallard/Sweden/80261/2008|H4N9
A/mallard duck/Netherlands/3/2008|H6N2
A/mallard/Yan chen/2005|H4N6
A/Eurasian teal/Netherlands/1/2008|H6N1
A/mallard duck/Netherlands/8/2008|H6N1
A/mallard/Sweden/99668/2009|H9N2
A/mallard/Sweden/99785/2009|H9N2
A/Bewicks swan/Netherlands/5/2007|H9N2
A/Eurasian wigeon/Netherlands/2/2008|H7N4
A/mallard duck/Netherlands/34/2006|H3N8
A/mallard/Netherlands/1/2006|H8N4
A/mallard/Sweden/6134/2005|H4N6
A/Anas crecca/Spain/1460/2008|H7N9
A/mallard/Sweden/52299/2006|H4N6
A/environment/Mongolia/1 33/2007|H4N6
A/mallard/Sweden/104925/2009|H4N6
A/mallard duck/Netherlands/23/2012|H11N9
A/ruddy shelduck/Mongolia/974/2010|H10N7
A/greater white fronted goose/Netherlands/4/2009|H5N2
A/greater white fronted goose/Netherlands/1/2010|H6N1
A/greater white fronted goose/Netherlands/3/2011|H6N2
A/mallard duck/Netherlands/29/2009|H11N9
A/chicken/Pakistan/NARC 16945/2010|H3N1
A/common shelduck/Mongolia/2076/2011|H3N8
A/canvasback/Xianghai/428/2011|H5N2
A/muscovy duck/Vietnam/LBM115/2012|H3N2
A/baikal teal/Xianghai/426/2011|H5N2
A/duck/Mongolia/146/2010|H3N6
A/aquatic bird/Korea/CN5/2009|H6N5
A/wild bird/Mongolia/2066/2011|H5N3
A/mallard duck/Netherlands/19/2009|H5N3
A/Eurasian wigeon/Netherlands/1/2010|H5N2
A/mallard duck/Netherlands/21/2010|H10N7
A/mallard duck/Netherlands/22/2010|H10N7
A/greater white fronted goose/Netherlands/4/2011|H1N1
A/duck/Bangladesh/31227/2016|H6N2
A/mallard/Republic of Georgia/1/2012|H1N1
A/duck/Mongolia/742/2015|H10N7
A/duck/Hubei/ZYSYG1/2015|H6N2
A/duck/Bangladesh/24035/2014|H10N1
A/hooded crane/Korea/1176/2016|H1N1
A/duck/Bangladesh/24692/2015|H7N1
A/duck/Bangladesh/24706/2015|H7N1
A/shorebird/Delaware Bay/250/2009 | A / H10N7
A/mallard/Arkansas/AI09-6032/2009 | A / H10N9
A/pintail duck/ALB/584/1984 | A / H10N6
A/shorebird/Delaware Bay/109/2001 | A / H10N7
A/mallard/Maryland/13OS3035/2014 | A / H10N7
A/northern shoveler/Washington/44249-700/2006 | A / H10N1
A/mallard/Ohio/11OS1966/2011 | A / H10N3
A/American green-winged teal/Mississippi/12OS405/2012 | A / H10N7
Eurasian lineage
North America lineage
0.005
